# Lactoferrin/κ-Carrageenan Interactions: Thermodynamic and Kinetic Insights through Surface Plasmon Resonance and Steady-State Fluorescence

**DOI:** 10.1021/acsomega.6c08383

**Published:** 2026-09-26

**Authors:** Yara Luiza Coelho Zampier, Isabela A. Marques, Álvaro Javier Patiño-Agudelo, Hauster Maximiler C. de Paula, Eliara A. Hudson, Ana Clarissa S. Pires, Luis Henrique M. da Silva

**Affiliations:** † Advanced Thermokinetics of Molecular Systems (ATOMS) Group, Chemistry Department, Federal University of Viçosa, PH Rolfs Avenue, 36570-000 Viçosa-MG, Brazil; ‡ Department of Food Technology, Federal University of Viçosa, Av. P. H. Rolfs s/s, 36570900 Viçosa-MG, Brazil

## Abstract

Protein–polysaccharide complexes play important roles in a wide range of technological applications. To elucidate the molecular interactions governing the formation of bovine lactoferrin (BLF)−κ-carrageenan (κCG) bionanostructures, the thermodynamic and kinetic parameters of BLF−κCG binding were determined using Steady-state fluorescence (FS) and surface plasmon resonance (SPR) spectroscopies. Formation of a 1:1 BLF-κCG complex is thermodynamically favorable at equilibrium (−47.00 ≤ Δ*G*_*FS*_^*o*^ ≤ −46.15 kJ mol^–1^) and is predominantly enthalpy-driven (Δ*H*_*FS*_^*o*^ = −57.43 kJ mol^–1^). Kinetic analysis by SPR revealed that the interaction proceeds through a transition complex ([BLF – κCG]^‡^), with complex formation occurring more rapidly through association of the free molecules (32.53 ≤ Δ*G*_*a*_^‡^ ≤ 38.52 kJ mol^–1^) than through dissociation of the thermodynamically stable complex (77.61 ≤ Δ*G*_*d*_^‡^ ≤ 81.62 kJ mol^–1^). Consistent with these findings, the energetic barrier for dissociation (*E*_*d*–298.2 *K*_^‡^ = 96.46 kJ mol^–1^) was higher than for association (*E*_*a*–298.2 *K*_^‡^ = 42.88 kJ mol^–1^). Increasing the ionic strength altered the BLF−κCG interaction mechanism, converting the binding process from a multistep pathway to a single-step process.

## Graphical abstract

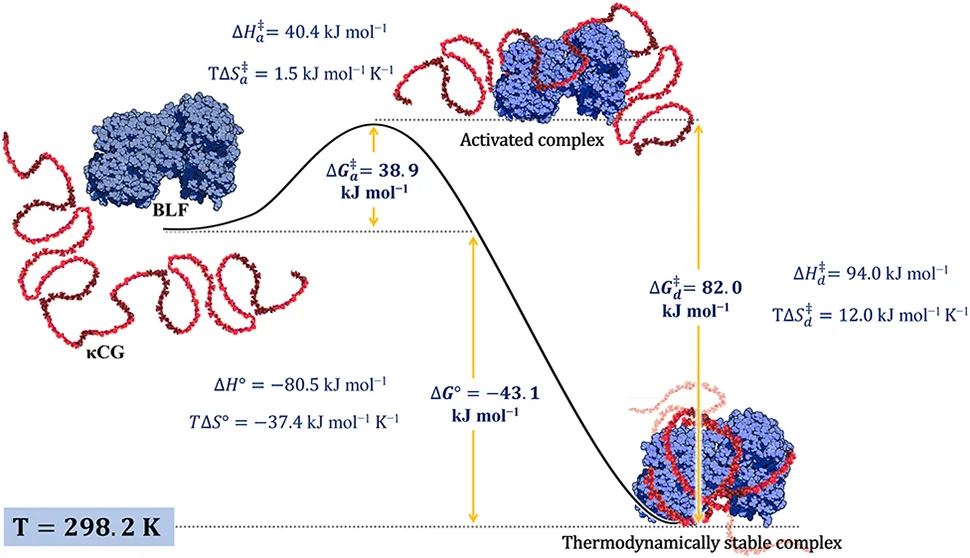

## 1 Introduction

Protein–polysaccharide interactions have attracted growing interest in recent years because of their broad applications in the food, pharmaceutical, cosmetic, and textile industries.^1–3^ These complexes have been extensively explored for drug delivery systems and the development of functional foods with enhanced nutritional value.^4–7^ Despite these advances, several fundamental aspects of protein–polysaccharide association remain poorly understood. For example, what are the relative contributions of electrostatic and dispersive interactions, including hydrogen bonding, hydrophobic interactions, and van der Waals forces, to complex formation? How do conformational changes influence molecular association and the dissociation of thermodynamically stable complexes? What role does molecular desolvation play during complexation? Finally, to what extent do ions within the electrical double layer contribute to the association and dissociation processes?

Addressing these questions is essential for elucidating the molecular basis of protein–polysaccharide interactions and identifying new nutraceutical properties and technological applications for these systems. Such understanding is particularly important because proteins and polysaccharides are ubiquitous components of biological systems and participate in numerous biochemical processes that are fundamental to life.^8,9^

The association of proteins and polysaccharides in aqueous solution can generate a variety of supramolecular structures, including water-soluble complexes, water-insoluble aggregates,^10^ and coacervates,^11^ depending on the balance between polymer–polymer and polymer–solvent interactions. Consequently, environmental factors such as pH, ionic strength, and the protein-to-polysaccharide ratio play decisive roles in governing the formation, stability, and physicochemical properties of these assemblies.^12,13^

Among the polysaccharides employed to prepare functional protein-based complexes, κ-carrageenan (κCG) has emerged as a particularly promising candidate because of its excellent gelling, thickening, and emulsifying properties, which have led to its widespread use in food applications.^14–16^ Furthermore, its biocompatibility, biodegradability, water solubility, and low toxicity make κCG an attractive material for the encapsulation and delivery of nutraceutical compounds.^17–20^ Structurally, κCG is a sulfated polysaccharide extracted from the cell walls of red algae and consists of alternating (1→3)-α-D-galactose-4-sulfate and (1→4)-β-3,6-anhydro-D-galactose repeating units.^21,22^ Although the interactions of κCG with several proteins, including serum albumin,^23,24^ casein,^25–27^ β-lactoglobulin,^28,29^ and lysozyme,^30^ have been extensively investigated, studies addressing its interaction with bovine lactoferrin (BLF) remain limited, and the field is still in its early stages.^31,32^

BLF is a cationic glycoprotein with an isoelectric point of approximately 8.7, a molecular mass of about 80 kDa, and nearly 700 amino acid residues. Its tertiary structure comprises two homologous globular lobes (N and C), each capable of binding one Fe^2+^ or Fe^3+^ ion.^33,34^ BLF performs diverse biological functions and exhibits remarkable immunomodulatory, antimicrobial, antiviral, antifungal, antitumor, and anti-inflammatory activities.^35^ These attributes make BLF an attractive ingredient for the development of functional and nutraceutical foods.^36^ Its versatility arises not only from its high affinity for iron but also from its ability to interact with a wide variety of biomolecules, including polysaccharides.

Indeed, BLF is readily associated with several polysaccharides, including pectin,^37^ gum Arabic,^38^ carboxymethylcellulose,^39^ and sodium alginate,^40^ yielding complexes with physicochemical and functional properties that differ markedly from those of the isolated components. As a result, these systems have been widely explored to enhance the techno-functional performance of food and nutraceutical formulations.^41^ For example, lactoferrin-polysaccharide complexes have proven to be efficient carriers for the oral delivery of nutraceuticals and pharmaceuticals while exhibiting greater thermal stability than the free protein. Moreover, they have shown considerable potential for micro- and nanoencapsulation, multilayer assembly, food emulsion stabilization, and the development of novel gel-based materials.^42^

Despite their technological relevance, the molecular mechanisms governing BLF–polysaccharide association remain incompletely understood. In particular, quantitative information regarding the thermodynamic driving forces, kinetic pathways, and the influence of electrolyte ions on complex formation is still scarce. To address these knowledge gaps, we performed a systematic thermodynamic and kinetic investigation of the interaction between BLF and κCG in the absence and presence of KCl. The interaction was investigated at pH 4.0, where BLF is positively charged, providing a relevant model condition for evaluating the contribution of electrostatic interactions to BLF−κCG association. Steady-state fluorescence spectroscopy (FS) and surface plasmon resonance (SPR) were employed to determine the binding constant (*K*_*b*_), standard Gibbs free energy change (Δ*G* ^*o*^), standard enthalpy change (Δ*H* ^*o*^), and standard entropy change (Δ*S* ^*o*^) of binding, together with the association (*k*_*a*_) and dissociation (*k*_*d*_) rate constants, activation energy (*E* ^‡^), and the Gibbs free energy (Δ*G* ^‡^), enthalpy (Δ*H* ^‡^), and entropy (*T*Δ*S* ^‡^) of activation. These findings provide new insights into the molecular recognition mechanism underlying BLF−κCG complexation and clarify the role of electrolyte ions in modulating this process.

## 2 Material and Methods

### 2.1 Materials

κ-carrageenan (κCG, product code 22048, quality level 100), bovine milk lactoferrin (BLF, ≥85 wt %), sodium acetate (CH_3_COONa, analytical grade), acetic acid (CH_3_CO_2_H, analytical grade), and potassium chloride (KCl, >99 wt %) were purchased from Sigma-Aldrich (St. Louis, MO, USA). Research-grade CM5 sensor chips and coupling reagents *N*-ethyl-*N*′-(dimethylaminopropyl)­carbodiimide hydrochloride (EDC), *N*-hydroxysuccinimide (NHS), and ethanolamine hydrochloride (1 M, pH 8.5) were purchased from GE Healthcare (Pittsburgh, PA, USA). The BLF used in this study was previously characterized by Pryshchepa et al.,^43^ who reported an iron content of 1.45 mg Fe g^–1^ protein and an isoelectric point of 7.4.

The commercial κCG used in this study was previously characterized by Eha et al.^44^ and Robal et al.^45^ The monosaccharide composition and sulfate content were determined by high-performance liquid chromatography and the BaCl_2_-gelatin turbidity method, respectively, following polysaccharide hydrolysis. The sample exhibited the following composition (dry weight basis): sucrose (26.3 ± 0.5 wt %), galactose (26.3 ± 0.4 wt %), 3,6-anhydro-D-galactose (25.4 ± 0.5 wt %), and sulfate (15.7 ± 0.3 wt %). The chemical structure was further confirmed by ^13^C NMR spectroscopy, which showed characteristic signals corresponding to (1→3)-α-D-galactose-4-sulfate [δ (ppm): 102.5 (C1), 59.5 (C2), 78.7 (C3), 74.0 (C4), 74.8 (C5), and 61.3 (C6)] and (1→4)-β-3,6-anhydro-D-galactose [δ (ppm): 95.0 (C1), 69.8 (C2), 79.2 (C3), 78.3 (C4), 76.8 (C5), and 69.5 (C6)]. The weight-average molecular weight (*M*_w_), determined by size-exclusion chromatography, was 1460 kDa. The mineral composition of the dry κCG sample was 0.59 wt % Na, 6.15 wt % K, 0.16 wt % Mg, 2.71 wt % Ca, and 7.19 wt % S.

### 2.2 Preparation of Biopolymer Solutions

BLF and κCG powders were weighed into separate beakers, followed by the addition of acetate buffer (pH 4.0). The BLF solutions were gently stirred at room temperature until complete dissolution and subsequently filtered through a 0.45 μm Millipore membrane filter. The real BLF concentration was determined spectrophotometrically using a Shimadzu UV-2550 double-beam spectrophotometer. The κCG solutions were heated at 343.2 K for 30 min and then stirred continuously for 24 h until a clear, homogeneous solution free of visible precipitates was obtained.

### 2.3 Steady-State Fluorescence Spectroscopy (FS) Measurements

FS measurements were performed using a Cary Eclipse fluorescence spectrophotometer (Agilent Technologies, Santa Clara, CA, USA) equipped with a thermostatic cell holder and 1 cm path-length quartz cuvettes. The experiments were conducted at 288.2, 293.2, 298.2, 303.2, and 313.2 K. Emission spectra were recorded from 296 to 500 nm following excitation of the tryptophan (Trp) residues of BLF at 295 nm, using excitation and emission slit widths of 2.5 and 5.0 nm, respectively, a photomultiplier tube (PMT) voltage of 750 V, and a scan rate of 600 nm min^–1^. Fluorescence spectra were acquired at a fixed BLF concentration of 19.5 μM and varying κCG concentrations ranging from 0 to 30.7 nM. Given the very low κCG concentrations used, no significant contribution of κCG to the absorbance at the excitation wavelength (295 nm) was expected, and no inner-filter correction was applied. Appropriate buffer-background controls were performed under the same experimental conditions, and the corresponding background signals were evaluated and found not to significantly affect the fluorescence measurements.

### 2.4 Surface Plasmon Resonance (SPR) Measurements

SPR experiments were performed using a Biacore X100 instrument (GE Healthcare, Pittsburgh, PA, USA) to determine the kinetic and thermodynamic parameters governing the BLF−κCG interaction. Prior to protein immobilization, CM5 sensor chips were activated with a mixture of EDC/NHS for 7 min, followed by blocking of the remaining activated carboxyl groups with ethanolamine for an additional 7 min. BLF (15 μg mL^–1^) prepared in 10 mM sodium acetate buffer (pH 4.0) was then immobilized on the sensor surface at a low immobilization level (3864 RU) to minimize mass transport limitations and steric crowding effects. A reference flow cell was prepared following the same activation and blocking procedure, but without BLF immobilization, to correct for nonspecific signals, instrument drift, and bulk refractive index changes. κCG solutions (15–28 nM) were injected at a flow rate of 30 μL min^–1^ for 90 s, followed by a dissociation period of 134 s. A sensorgram was recorded for each κCG concentration under each experimental condition.

Binding experiments were performed at temperatures ranging from 285.2 to 301.2 K. For each experiment, κCG solutions were injected over both the reference and BLF-immobilized flow cells at the desired concentration. Prior to each binding cycle, acetate buffer (pH 4.0) was injected to establish a stable baseline. A buffer-only blank injection was also performed for each κCG concentration series to account for any response associated with the running buffer.

### 2.5 Dynamic Light Scattering (DLS) and Zeta Potential (ζ) Measurements

The hydrodynamic diameter (*D*_*H*_) and zeta potential (ζ) of free BLF, free κCG, and BLF−κCG mixtures containing different κCG concentrations were measured at 298.2 K using a Zetasizer Nano ZS (Malvern Instruments, Malvern, UK) equipped with a 633 nm He–Ne laser operating at 4 mW.

## 3 Results and Discussion

### 3.1 Study of BLF−κCG Interactions Using Fluorescence Spectroscopy

A commonly used technique for studying the formation of protein-based complexes is FS, which, through the fluorescence quenching phenomenon, enables the determination of the binding constant (*K*_*b*_) and, indirectly, the thermodynamic parameters, including the standard Gibbs free energy change (Δ*G* ^*o*^), standard enthalpy change (Δ*H* ^*o*^), and standard entropy change (Δ*S* ^*o*^).^46,47^

Trp residues are the primary intrinsic fluorophores in proteins. When a molecule binds in the vicinity of a Trp residue (typically within ∼ 5 Å), changes in the intrinsic fluorescence can be observed.^48^ Therefore, to gain insight into the effect of κCG on the Trp microenvironment in native BLF at pH 4.0, the intrinsic fluorescence of BLF was monitored upon excitation at 295 nm in the presence of increasing κCG concentrations (Figure 1).

**Figure 1:**
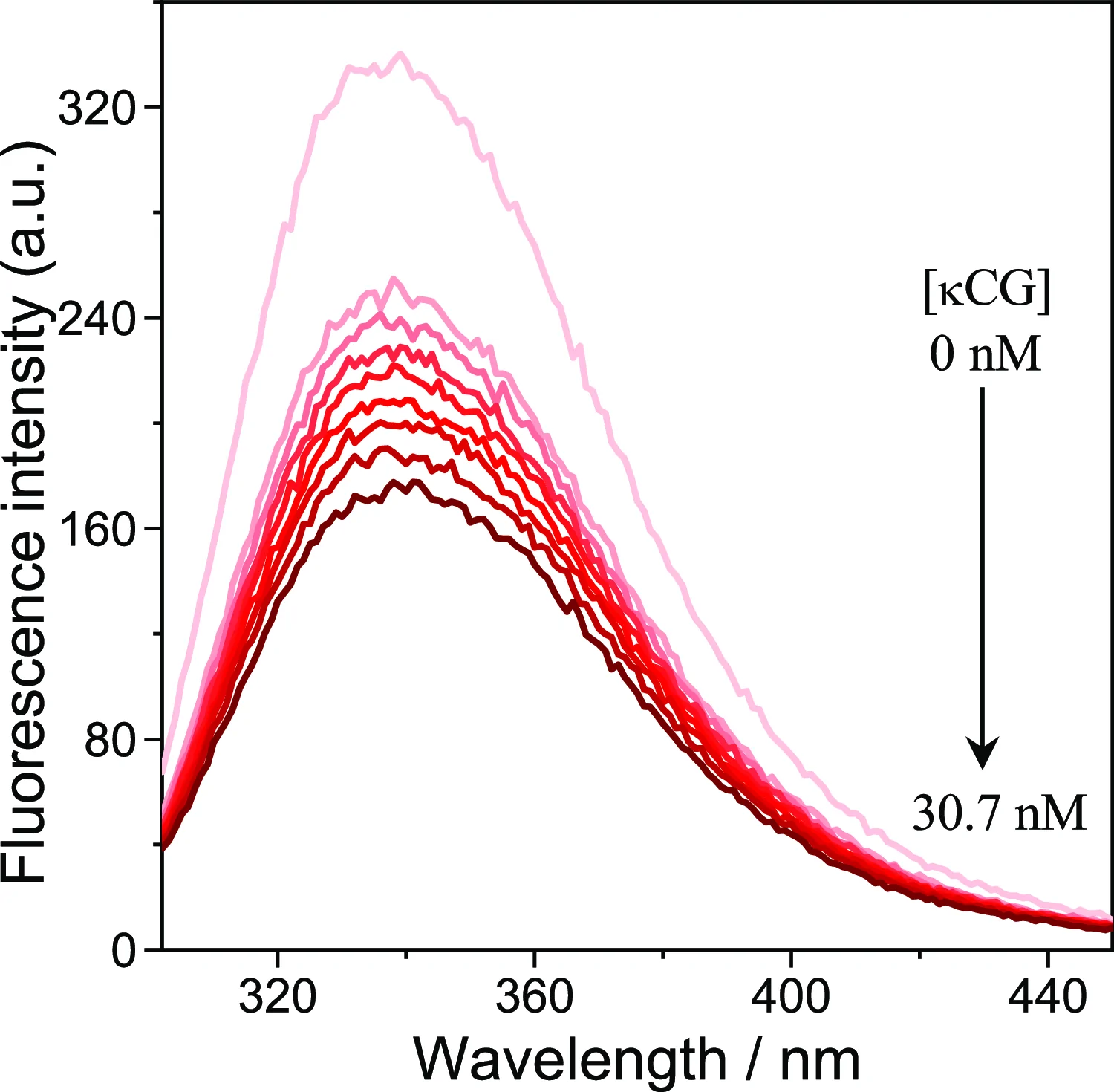
Steady-state fluorescence emission spectra of native BLF (19.5 μM) in buffer at pH 4.0 and 298.2 K in the presence of increasing κCG concentrations (0–30.7 nM).

The fluorescence intensity of native BLF at 336 nm decreased with increasing κCG concentration. No shift in the maximum emission wavelength was observed, suggesting that κCG binding did not induce a substantial change in the local environment of the Trp residues detectable by fluorescence spectroscopy. Given that most Trp residues are located within hydrophobic regions of the protein, the absence of a spectral shift may indicate that no major alteration in the tertiary structure around these residues occurred upon κCG binding.^49^ However, this observation alone does not exclude more subtle conformational changes in BLF, which would require complementary structural techniques for confirmation.

A decrease in fluorescence intensity may result from several processes, including molecular rearrangement, energy transfer, ground-state complex formation, and collisional quenching.^50^ Among these, static and dynamic quenching are the most common mechanisms. Different models have been proposed to interpret fluorescence quenching data and thereby provide insight into the mechanism responsible for the observed decrease in fluorescence intensity.^51^ In this study, the Stern–Volmer model^52^ (eq 1) was employed to determine whether the quenching mechanism was static or dynamic.

1 $$\frac{F_{0}}{F}=1+K_{SV}\left[\kappa CG\right]=1+k_{q}\tau_{0}\left[\kappa CG\right]$$

Here, *F*_0_ and *F* are the fluorescence intensities of the fluorophore (BLF) in the absence and presence of the quencher (κCG), respectively, *K*_SV_ is the Stern–Volmer quenching constant, [κCG] is the quencher concentration, *k*_*q*_ is the bimolecular quenching rate constant, and τ_0_ is the average fluorescence lifetime of BLF in the absence of the quencher (1.06 ns).^53^

The Stern–Volmer plots for the quenching of native BLF by κCG at 288.2, 293.2, 298.2, 303.2, and 313.2 K are shown in Figure S1a. The corresponding *K*_SV_ and *k*_*q*_ values, obtained from the slopes of the plots, are listed in Table S1. The *K*_SV_ values increased with increasing temperature up to 298.2 K (0.66–2.63 × 10^7^ M^–1^) and then decreased at temperatures above 303.2 K (2.47–1.93 × 10^7^ M^–1^). The *k*_*q*_ values (0.62–1.82 × 10^16^ M^–1^ s^–1^) exceeded the maximum diffusion-controlled quenching rate constat (2.0 × 10^10^ M^–1^ s^–1^), indicating that fluorescence quenching occurred predominantly through static mechanism, i.e., via ground-state complex formation between BLF and κCG.^48^ Accordingly, *K*_*b*_ and the number of binding sites (*n*) were determined from the intercept and slope, respectively, of the double-logarithmic plot based on eq 2,^54^ as shown in Figure S1b.

2 $$\log\left(\frac{F_{0}‐F}{F}\right)=\log K_{b}+n\log\left[\kappa CG\right]$$

Here, *n* is the number of ligand molecules bound per protein molecule, and [κCG] is the total ligand concentration. The corresponding *K*_b_ and *n* values are summarized in Table 1. The *K*_*b*_^FS^ values were on the order of 10^8^ M^–1^ and decreased with increasing temperature, indicating that complex formation is an exothermic process, as commonly observed for protein-based systems.^55^ Moreover, the *n* values were close to 1 at all temperatures, suggesting an apparent 1:1 binding stoichiometry between BLF and κCG.

**Table 1:** Binding Constant (*K*_*b*_^*FS*^), Apparent Number of Binding Sites (*n*), and Thermodynamic Parameters (Δ*G*_FS_^*o*^, Δ*H*_FS_^*o*^, and *T*Δ*S*_*FS*_^*o*^) for BLF−κCG Complex Formation at Different Temperatures (*T*) and pH 4.0^a^

***T***	***K***_***b***_^**FS**^	***n***	Δ ***G***_**FS**_^***o***^	Δ ***H***_**FS**_^***o***^	***T*** Δ ***S***_**FS**_^***o***^
K	10^8^ M^–1^		kJ mol^–1^		
288.2	3.3 ± 0.1	1.2 ± 0.1	–47.0 ± 0.1	–57.4 ± 0.1	–10.4 ± 0.1
293.2	2.2 ± 0.1	1.2 ± 0.1	–46.8 ± 0.1	–57.4 ± 0.1	–10.6 ± 0.1
298.2	1.5 ± 0.1	1.1 ± 0.1	–46.7 ± 0.2	–57.4 ± 0.1	–10.7 ± 0.2
303.2	1.0 ± 0.1	1.1 ± 0.1	–46.3 ± 0.4	–57.4 ± 0.1	–11.1 ± 0.4
313.2	0.5 ± 0.1	1.1 ± 0.1	–46.2 ± 0.5	–57.4 ± 0.1	–11.3 ± 0.5

a Data obtained from fluorescence measurements.

To investigate the size and surface charge of the complexes formed, the *D*_*H*_ and ζ of individual components (BLF and κCG), as well as of the thermodynamically stable BLF-κCG complexes, were measured by DLS. The results are summarized in Table S2. At pH 4.0 and 298.2 K, free BLF and κCG exhibited positive Zeta potentials (ζ_BLF_ = 14 ± 2 mV and ζ_κCG_ = 5 ± 1 mV) and hydrodynamic diameters of 72 ± 1 and 503 ± 54 nm, respectively. In contrast, BLF-κCG mixtures containing different κCG concentrations exhibited ζ values ranging from 20 to 23 mV and *D*_*H*_ values between 143 and 278 nm.

Although κCG contains negatively charged sulfate groups, the positive ζ-potential measured at pH 4.0 may reflect the electrokinetic behavior of the commercial material rather than the intrinsic charge of its polysaccharide backbone. The κCG used in this study was a commercial product with a reported ratio of positively to negatively charged species of approximately 1.48, indicating a predominance of cationic species.^45^ The associated cations can modify the electrical double layer surrounding the polysaccharide and consequently influence the measured ζ-potential.^56^ Under the experimental conditions, the anionic sulfate groups of κCG may therefore contribute to the interaction with positively charged regions of BLF through electrostatic attraction.

These findings indicate that electrostatic attraction between positively charged regions of BLF and negatively charged sulfate groups of κCG, together with other intermolecular interactions, contributes to the formation of positively charged complexes. The intermediate hydrodynamic diameters suggest that complex formation is accompanied by conformational rearrangements, likely involving partial wrapping of the κCG chains around BLF molecules.

Protein–polysaccharide interactions are typically governed by hydrogen bonding, hydrophobic interactions, electrostatic interactions, and van der Waals forces.^57^ The signs and magnitudes of the thermodynamic parameters provide valuable insight into the intermolecular forces driving complex formation and, consequently, the underlying binding mechanism. Based on the *K*_*b*_^FS^ values, Δ*G*° was calculated using eq 3:

3 $$\Delta G^{{}^{\circ}}=-\mathrm{RT}\;\ln K_{b}$$

where *R* is the universal gas constant (8.314 J mol K^–1^) and *T* is the absolute temperature (K). The standard enthalpy change (Δ*H*°) was calculated using the van’t Hoff equation (eq 4) from the slope of the ln *K*_*b*_^FS^ versus 1/*T* plot (Figure S2).


4 $$\ln\left(\frac{K_{b}T_{2}}{K_{b}T_{1}}\right)=-\frac{\Delta H^{{}^{\circ}}}{R}\left(\frac{1}{T_{2}}-\frac{1}{T_{1}}\right)$$

Using the obtained Δ*H*° and Δ*G*° values, the standard entropy change (Δ*S*°) was calculated through the fundamental Gibbs equation (eq 5).

5 $$\Delta G^{{}^{\circ}}=\Delta H^{{}^{\circ}}-T\Delta S^{{}^{\circ}}$$

The thermodynamic parameters for complex formation at all studied temperatures are listed in Table 1. The Δ*G*_FS_^*o*^ values were all negative and increased with increasing temperature, indicating that, in the system, the complex predominated over the free molecules and that the BLF−κCG complexes became less stable as the temperature increased. Overall, these values result from the contributions of the different processes occurring in the system; thus, to better understand these processes, the enthalpic and entropic contributions were analyzed.

The Δ*H*_FS_^*o*^ value was negative, suggesting that the energy released upon the formation of new intermolecular interactions between BLF and κCG outweighs the energetic cost associated with desolvation and, in particular, the release of counterions from the electric double layers surrounding the interacting molecules. Similarly, the *T*Δ*S*_FS_^*o*^ values were negative. Therefore, the entropic gain resulting from the desolvation of free BLF and κCG molecules and the release of counterions from the electric double layers is outweighed by the loss of configurational entropy accompanying BLF−κCG complex formation.

### 3.2 Kinetics and Thermodynamics of BLF−κCG Complex Formation Determined by Surface Plasmon Resonance

#### 3.2.1 Kinetic Study

Although the thermodynamic parameters provide important information about the energetics involved in protein–polysaccharide complex formation, kinetic studies are indispensable for understanding the molecular processes that occur during the interaction between these species. In contrast to thermodynamic parameters obtained from equilibrium data, nonequilibrium kinetic parameters provide information that extends beyond the bound and unbound states.^58,59^

SPR-based biosensors are widely used to investigate the interaction kinetics between macromolecules.^60–62^ This optical technique detects changes in the refractive index within approximately 100 nm of the sensor surface, where the ligand (BLF, in the present study) is immobilized. When the analyte (κCG) binds to the immobilized ligand, the local refractive index changes, allowing the interaction to be monitored in real time. The resulting response is recorded as a sensorgram, i.e., a plot of resonance units (RU) versus time, as shown in Figure 2 for the BLF−κCG interaction at 298.2 K and pH 4.0.

**Figure 2:**
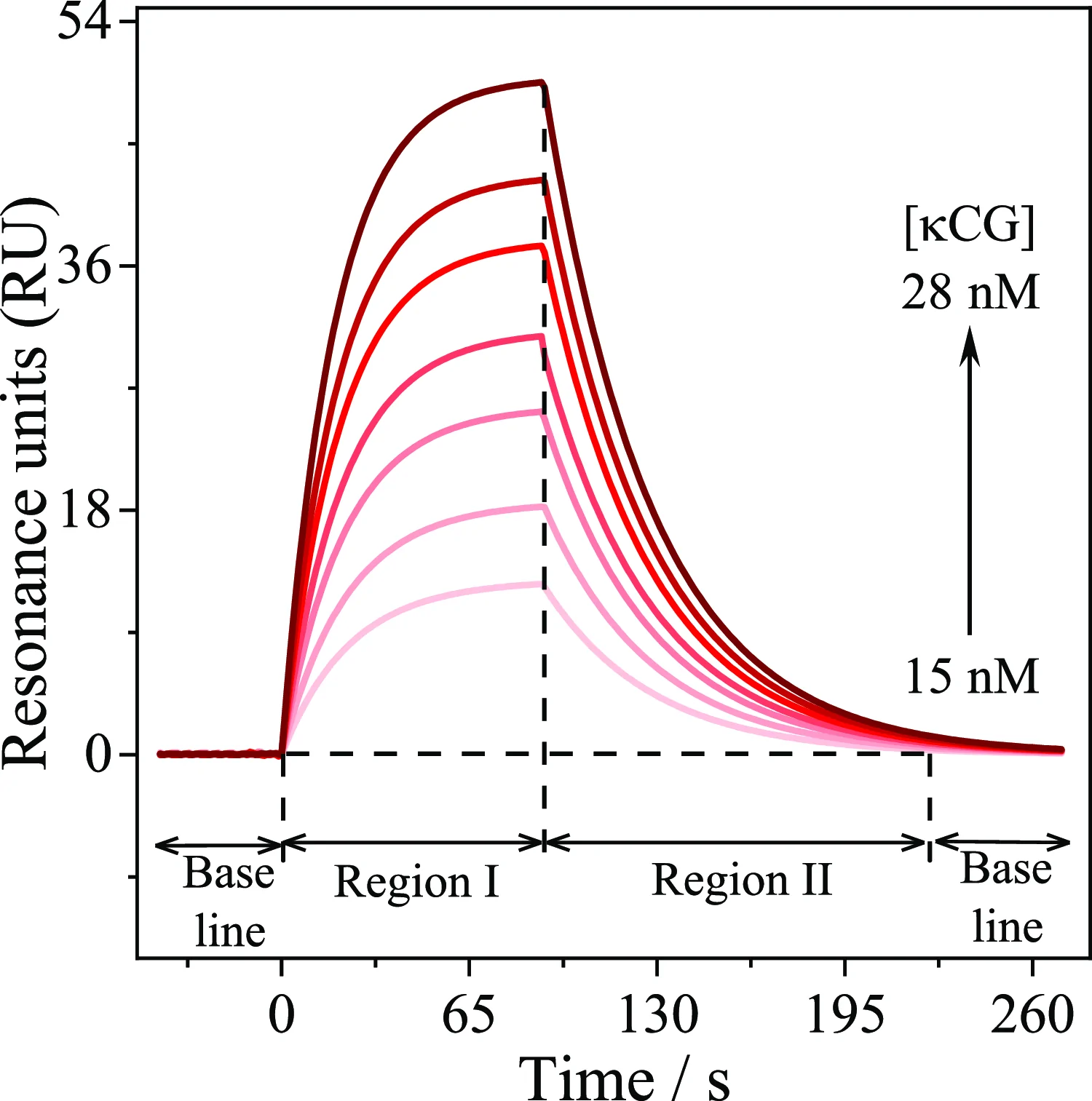
Representative SPR sensorgrams (RU versus time) for the interaction between BLF and κCG obtaining by injecting κCG solutions (15–28 nM) over a CM5 sensor chip with low-density immobilized BLF (3864 RU) at 298.2 K and pH 4.0. The arrow indicates increasing κCG concentration. Sensorgrams recorded at the other temperatures are shown in Figure S3.

The SPR data demonstrates that, although BLF is immobilized on the sensor surface, it retains its ability to form thermodynamically stable complexes with κCG. The sensorgrams exhibit two distinct regions: (I) an ascending phase corresponding to association and (II) a descending phase corresponding to dissociation. During region I (0–90 s), the association of free κCG with immobilized BLF and the dissociation of thermodynamically stable BLF−κCG complexes occur simultaneously, with association predominating at the beginning of the ascending phase. In region II (90–224 s), dissociation becomes the predominant process.

To determine the kinetic parameters governing BLF−κCG complex formation, namely the association (*k*_*a*_) and dissociation (*k*_*d*_) rate constants, the sensorgrams were globally fitted to a 1:1 binding model (eqs 6 and 7). The resulting values are listed in Table 2.

6 $$\mathrm{RU}\left(t\right)=\mathrm{RU}\left(t_{m}\right)e^{-k_{d}\left(t-t_{m}\right)}$$

7 $$\mathrm{RU}\left(t\right)={\mathrm{RU}}_{\max}\left(t_{\infty }\right)\left[1-e^{-k_{obs}\left(t\right)}\right]$$

Here, *RU*(*t*) is the SPR response at time *t*, *RU*(*t*_*m*_) is the SPR response at the onset of the dissociation phase of the sensorgram, *k*_obs_ is the observed rate constant, and [*RU*_max_(*t*_*∞*_)] is the resonance signal at infinite time (*t*_*∞*_). Because *k*_obs_ = *k*_*a*_ [κCG] + *k*_*d*_, the *k*_*a*_ values were determined from the slope of the *k*_obs_ versus [κCG] plots (Figure S4).

**Table 2:** Association (*k*_*a*_) and Dissociation (*k*_*d*_) Rate Constants for BLF−κCG Complex Formation at Different Temperatures and pH 4.0

***T***	***k***_***a***_	***k***_***d***_
K	10^6^ M^–1^ s^–1^	10^–2^ s^–1^
285.2	6.5 ± 0.1	0.036 ± 0.001
289.2	2.0 ± 0.1	0.022 ± 0.001
293.2	1.1 ± 0.1	0.019 ± 0.001
297.2	0.9 ± 0.1	0.023 ± 0.001
298.2	1.0 ± 0.1	0.027 ± 0.001
301.2	1.3 ± 0.1	0.044 ± 0.001

To date, there have been no reports on the kinetic of the interaction between BLF and κCG. However, Coelho et al. investigated the interaction between BLF and the polysaccharide carboxymethylcellulose using SPR.^39^ At 298.2 K, they reported an association rate constant approximately 1 order of magnitude lower (*k*_*a*_ = 1.8 × 10^5^ M^–1^s^–1^) and a dissociation rate constant approximately 2 orders of magnitude higher (*k*_*d*_ = 6.4 × 10^–2^ s^–1^) than those obtained here for the BLF−κCG complex (*k*_*a*_ = 1.0 × 10^6^ M^–1^s^–1^ and *k_d_* = 2.7 × 10^–4^ s^–1^).

Studying the energetics of transition (or activated) complex formation ([BLF – κCG]^‡^) can provide additional information about the binding of κCG to BLF. This approach enables a detailed analysis of the energy landscape associated with the association of the free species and the dissociation of the thermodynamically stable complex, as well as the determination of the activation energy barrier separating the bound and unbound states.^63^ Therefore, Arrhenius plots (Figure 3) were constructed to determine the energetic parameters associated with the formation of [BLF – κCG]^‡^ through the association (*a*) of the free molecules (κCG and BLF) and the dissociation (*d*) of the thermodynamically stable BLF−κCG complex.

**Figure 3:**
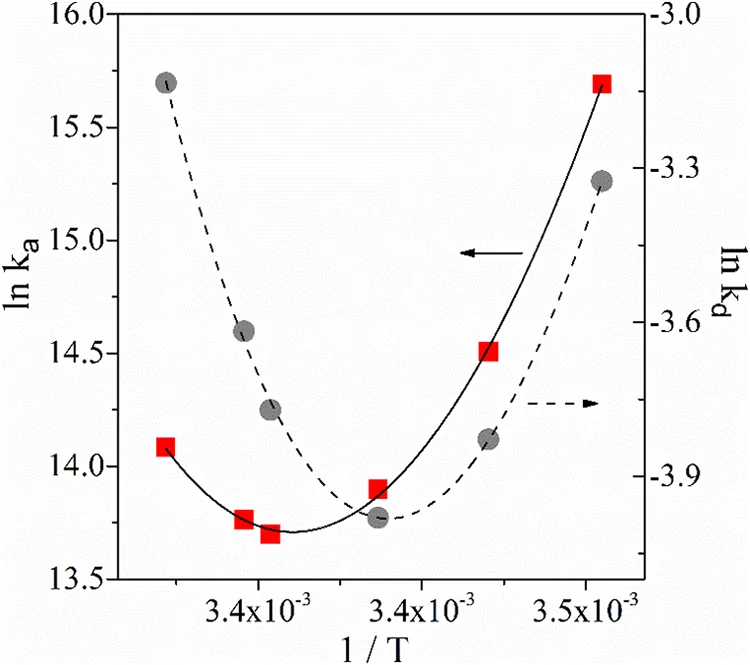
Arrhenius plots of ln *k*_*a*_ (■) and ln *k*_*d*_ (●) versus (1/*T*) for the BLF−κCG interaction at pH 4.0.

Both *ln k*_*a*_ and *ln k*_*d*_ exhibited a polynomial dependence on 1/*T*, indicating that the energy barrier for the formation of the transition complex, [BLF – κCG]^‡^, is temperature-dependent over the investigated temperature range. This nonlinearity in the Arrhenius plots suggests that the formation of the transition complex, whether through the association of the free BLF and κCG macromolecules or through dissociation of the thermodynamically stable BLF-κCG complex, proceeds via a multistep molecular mechanism.^64^

The activation energies (*E*_*x*_^‡^) were determined using a nonlinear Arrhenius analysis (Figure 3) by fitting eqs 8 and 9. The remaining activation parameters, namely the Gibbs free energy change of activation (Δ*G*_*x*_^‡^), enthalpy of activation (Δ*H*_*x*_^‡^), and entropy of activation (Δ*S*_*x*_^‡^) were calculated using eqs 10–12, respectively. The resulting values are listed in Table 3.

8 $$\ln k_{x}=a+b\left(\frac{1}{T}\right)+c{\left(\frac{1}{T}\right)}^{2}+d{\left(\frac{1}{T}\right)}^{3}$$

9 $$E_{x}^{‡}=-R\left[b+2c\left(\frac{1}{T}\right)+3d{\left(\frac{1}{T}\right)}^{2}\right]$$

10 $$\Delta G_{x}^{‡}=-\mathrm{RT}\ln\left(\frac{k_{x}h}{k_{B}T}\right)$$

11 $$\Delta H_{x}^{‡}=E_{x}^{‡}-\mathrm{RT}$$

12 $$T{\Delta S}_{x}^{‡}={\Delta H}_{x}^{‡}-{\Delta G}_{x}^{‡}$$

Here, *b*, *c*, and *d* are constants obtained from the polynomial fit shown in Figure 3, the subscript “*x*” indicates association (*a*) or dissociation (*d*) process, and *R*, *k*_*B*_, and *h* are the universal gas, Boltzmann, and Planck constants, respectively.

**Table 3:** Activation Parameters for the Formation of the Transition Complex Through (*a*) Association of Free BLF and κCG Molecules and (*d*) Dissociation of the Thermodynamically Stable BLF−κCG Complex at Different Temperatures and pH 4.0, in the Absence and Presence of 100 mM KCl^a^

***T***	**association step (** *a* **)**				**dissociation step (** *d* **)**			
	***E*_*a*_^‡^**	**Δ** *H*_*a*_^**‡**^	**Δ** *G*_*a*_^**‡**^	*T* **Δ** *S*_*a*_^**‡**^	***E*_*d*_^‡^**	**Δ** *H*_*d*_^**‡**^	**Δ** *G*_*d*_^**‡**^	*T* **Δ** *S*_*d*_^**‡**^
**K**	kJ mol^–1^							
**0 mM KCl**								
285.2	–238.8 ± 0.9	–241.2 ± 0.9	32.5 ± 0.1	–273.7 ± 0.8	–113.5 ± 0.1	–115.8 ± 0.1	77.6 ± 0.1	–193.5 ± 0.1
289.2	–158.1 ± 0.9	–160.5 ± 0.9	35.9 ± 0.1	–196.7 ± 0.8	–57.8 ± 0.1	–60.2 ± 0.1	79.9 ± 0.1	–140.1 ± 0.1
293.2	–71.9 ± 0.5	–74.3 ± 0.7	37.9 ± 0.1	–112.2 ± 0.7	6.1 ± 0.1	3.7 ± 0.1	81.5 ± 0.1	–77.8 ± 0.1
297.2	19.4 ± 0.1	16.9 ± 0.1	38.9 ± 0.1	–22.1 ± 0.1	77.5 ± 0.1	75.1 ± 0.1	82.1 ± 0.1	–7.0 ± 0.1
298.2	42.9 ± 0.1	40.4 ± 0.1	38.9 ± 0.1	1.5 ± 0.1	96.5 ± 0.1	94.0 ± 0.1	82.0 ± 0.1	12.0 ± 0.2
301.2	115.1 ± 0.9	112.6 ± 0.9	38.5 ± 0.1	74.1 ± 0.9	155.7 ± 0.7	153.2 ± 0.2	81.6 ± 0.1	71.6 ± 0.2
**100 mM KCl**								
285.2	–37.5 ± 0.9	–39.9 ± 0.3	34.3 ± 0.9	–74.2 ± 0.9	28.9 ± 0.8	26.6 ± 0.5	78.6 ± 0.9	–52.0 ± 0.9
289.2	–37.5 ± 0.9	–39.9 ± 0.5	35.4 ± 0.9	–75.3 ± 0.9	28.9 ± 0.8	26.5 ± 0.7	79.3 ± 0.9	–52.7 ± 0.9
293.2	–37.5 ± 0.9	–40.0 ± 0.5	36.5 ± 0.9	–76.4 ± 0.9	28.9 ± 0.8	26.5 ± 0.8	80.0 ± 0.9	–53.5 ± 0.9
297.2	–37.5 ± 0.9	–40.0 ± 0.5	37.3 ± 0.9	–77.3 ± 0.9	28.9 ± 0.8	26.5 ± 0.7	80.8 ± 0.9	–54.3 ± 0.9
298.2	–37.5 ± 0.9	–40.0 ± 0.5	37.7 ± 0.9	–77.7 ± 0.9	28.9 ± 0.8	26.5 ± 0.7	80.9 ± 0.9	–54.4 ± 0.9
301.2	–37.5 ± 0.9	–40.0 ± 0.5	38.6 ± 0.9	–78.6 ± 0.9	28.9 ± 0.8	26.4 ± 0.7	81.5 ± 0.9	–55.1 ± 0.9

a For 100 mM KCl, *E*_*a*_^‡^ and *E*_*d*_^‡^ were obtained from Arrhenius fits over the complete temperature range and are reported as single values.

The *E*_*x*_^‡^ and Δ*H*_*x*_^‡^ values increased with increasing temperature for both the association and dissociation processes. *E*_*a*_^‡^ and Δ*H*_*a*_^‡^ were negative below 293.2 K, whereas *E*_*d*_^‡^ and Δ*H*_*d*_^‡^ were negative below 289.2 K, becoming positive at higher temperatures. Moreover, at all temperatures, the activation energy barrier was higher for dissociation than for association. This barrier arises because existing intra- and intermolecular interactions must be disrupted while new interactions are simultaneously established as BLF and κCG interact to form the transition complex, [BLF – κCG]^‡^.

The *E*_*x*_^‡^ and Δ*H*_*x*_^‡^ values arise from the combined contributions of the following subprocesses: (I) the restructuring of the solvation shell surrounding the interacting sites of BLF and κCG (*E*_*x*_^‡^ and Δ*H*_*x*_^‡^ > 0 for desolvation and *E*_*x*_^‡^ and Δ*H*_*x*_^‡^ < 0 for solvation); (II) electrostatic attraction between BLF and κCG, accompanied by charge redistribution within the electrical double layer (*E*_*x*_^‡^ and Δ*H*_*x*_^‡^ < 0); (III) conformational changes at the macromolecular binding sites (*E*_*x*_^‡^ and Δ*H*_*x*_^‡^ > 0); and (IV) direct interactions between κCG and BLF binding sites (*E*_*x*_^‡^ and Δ*H*_*x*_^‡^ < 0).

Therefore, at low temperatures, the negative *E*_*x*_^‡^ and Δ*H*_*x*_^‡^ values indicate that the energetic contribution from direct BLF-κCG interactions (|II + IV|) outweighs the combined contribution from solvation restructuring, and conformational changes (|I + III|). In other words, the average collisional kinetic energy (3/2*k*_*B*_ *T*) is not sufficiently high for desolvation, counterion release from the electrical double layer, and conformational rearrangements of the protein and polysaccharide binding sites to become the dominant processes.

However, with increasing temperature, the average collisional kinetic energy becomes sufficiently high to overcome the energy barriers associated with ion confinement within the electrical double layer, conformational rearrangements of the binding sites, and the removal H_2_O molecules solvating the functional groups involved in the protein–polysaccharide interaction. As a result, the combined energetic contributions of processes |I + III| exceed that of process |II + IV|, leading to positive *E*_*x*_^‡^ and Δ*H*_*x*_^‡^ values.

The formation of the transition complex, [BLF – κCG]^‡^, through the association of free κCG and BLF macromolecules was found to be faster than its formation through dissociation of the thermodynamically stable complex (Δ*G*_*a*_^‡^ < Δ*G*_*d*_^‡^), probably because the energy barrier is lower for the association process (*E*_*a*_^‡^ and Δ*H*_*a*_^‡^ < *E*_*d*_^‡^ and Δ*H*_*d*_^‡^). However, in addition to Δ*H*_*x*_^‡^, the entropic contribution also influences Δ*G*_*x*_^‡^, highlighting the importance of analyzing the *T*Δ*S*_*x*_^‡^ term.

At low temperatures, the *T*Δ*S*_*x*_^‡^ values were negative for both processes (association and dissociation), becoming positive only at 298.2 K. Moreover, similarly to *E*_*x*_ and Δ*H*_*x*_^‡^, the *T*Δ*S*_*x*_^‡^ values were higher for the dissociation of the thermodynamically stable BLF−κCG complex than for the association of free BLF and κCG molecules.

The *T*Δ*S*_*x*_^‡^ values can also be interpreted as the sum of the sum of the contributions from four molecular subprocesses: (I) conformational changes at the protein and polysaccharide binding sites (*T*Δ*S*_*x*_^‡^ > 0), (II) desolvation of κCG and BLF (*T*Δ*S*_*x*_^‡^ > 0), (III) release of counterions from the electrical double layer surrounding the interacting macromolecules (*T*Δ*S*_*x*_^‡^ > 0), and (IV) formation of the BLF−κCG complex (*T*Δ*S*_*x*_^‡^ < 0). The negative *T*Δ*S*_*x*_^‡^ values observed at low temperatures suggest that |IV| > |I + II + III|, probably because, in this temperature range, [BLF – κCG]^‡^ formation occurs without the significant release of H_2_O molecules and counterions and with few conformational changes at the macromolecule interaction sites. These results can be attributed to the fact that at low temperatures, the κCG molecules bind to the BLF surface, thus maintaining the conformation of the binding sites and the solvation shell of both interacting macromolecules. However, with increasing temperature, *T*Δ*S*_*x*_^‡^ becomes positive because the higher average collisional kinetic energy (3/2*k*_*B*_ *T*) increases the flexibility of the BLF binding sites during the interaction (process I) and promotes the release of H_2_O molecules and counterions from the solvation shell and the electrical double layer (processes II and III), respectively. As a result, the combined positive entropic contributions of processes I–III exceed the configuration entropy loss associated with complex formation (process IV), i.e., |I + II + III| > |IV|.

##### 3.2.1.1 Effect of Ionic Strength on the Formation of the Transition Complex [BLF – κCG]^‡^

To understand how electrostatic interactions contribute to the formation of the transition-state complex, [BLF – κCG]^‡^, the effect of ionic strength on the BLF−κCG interaction was investigated. The sensorgrams for the BLF−κCG interaction in the presence of 100 mM KCl at 285.2–301.2 K are presented in Figure S5. The corresponding observed rate constants (*k*_obs_) are shown in Figure S6, while the calculated association (*k*_*a*_) and dissociation (*k*_*d*_) rate constants are listed in Table 4. Measurements were also performed at 1 and 10 mM KCl, with the corresponding parameters provided in Table S3 of the Supporting Information; these conditions were not further discussed due to their negligible effects relative to the KCl-free system.

**Table 4:** Association (*k*_*a*_) and Dissociation (*k*_*d*_) Rate Constants for the Interaction Between BLF and κCG in the Presence of 100 mM KCl at pH 4.0

***T***	***k***_***a***_	***k***_***d***_
K	10^6^ M^–1^ s^–1^	10^–2^ s^–1^
285.2	3.1 ± 0.1	0.024 ± 0.004
289.2	2.4 ± 0.1	0.029 ± 0.006
293.2	2.0 ± 0.1	0.034 ± 0.005
297.2	1.7 ± 0.1	0.039 ± 0.005
298.2	1.6 ± 0.1	0.042 ± 0.006
301.2	1.3 ± 0.1	0.046 ± 0.006

Compared with the values obtained in the absence of KCl (Table 2), the presence of KCl increased both *k*_*a*_ and *k*_*d*_ at all temperatures, except at 285.2 K, where the opposite trend was observed. A possible molecular mechanism underlying these changes in *k*_*a*_ and *k*_*d*_ is the shielding of the electrical double layer, which decreases the charge asymmetry in the interfacial region and promotes the release of counterions from both macromolecules as the ionic strength increases. These results indicate that, to understand how changes in ionic strength affect the kinetics of [BLF – κCG]^‡^ formation, it is essential to evaluate the effect of KCl on the activation parameters. The values of *E*_*x*_^‡^, Δ*G*_*x*_^‡^, Δ*H*_*x*_^‡^, and*T*Δ*S*_*x*_^‡^ provide valuable insights into the molecular energetics and dynamics associated with the BLF−κCG interaction and can be readily obtained from the temperature dependence of *k*_*a*_ and *k*_*d*_ (Figure S7 and Table 3).

In the absence of KCl, the BLF−κCG interaction involves multiple molecular processes, as evidenced by the temperature dependence of the *E*_*x*_^‡^ values.^64^ In contrast, after the addition of KCl, the interaction is characterized by a single activation process, with *E*_*x*_^‡^ for both association and dissociation becoming temperature-independent. These results suggest that the presence of salt reduces the contribution of complex molecular events, such as conformational changes and counterion release from the electrical double layer, thereby making the BLF−κCG interaction mechanistically more straightforward.

Although the driving force for [BLF – κCG]^‡^ formation (Δ*G*_*x*_^‡^) was not affected by changes in ionic strength, a pronounced effect was observed when its enthalpic (Δ*H*_*x*_^‡^) and entropic (TΔ*S*_*x*_^‡^) contributions were analyzed. In the presence of KCl, Δ*H*_*a*_^‡^ was negative at all temperatures (−40.0 ≤ Δ*H*_*a*_^‡^ ≤ −39.9 kJ mol^–1^), whereas Δ*H*_*d*_^‡^ was positive (26.4 ≤ Δ*H*_*d*_^‡^ ≤ 26.6 kJ mol^–1^). In contrast, TΔ*S*_*x*_^‡^ was negative for both association (−78.6 ≤ *T*Δ*S*_*a*_^‡^ ≤ −74.2 kJ mol^–1^) and dissociation (−55.1 ≤ *T*Δ*S*_*d*_^‡^ ≤ −52.0 kJ mol^–1^) steps.

In the association step, the negative Δ*H*_*a*_^‡^ values are likely associated with the shielding of the electrical double layer by the added salt, which decreases the electrostatic interactions within the protein and polysaccharide and the attractive interactions between the counterions in the electrical double layer and the biomolecular surfaces. As a result, the binding sites of both macromolecules become more flexible owing to the screening of electrostatic interactions within the biopolymers. Consequently, the energetic cost associated with binding-site conformational rearrangements and solvation-shell reorganization during the formation of [BLF – κCG]^‡^ is reduced, leading to negative Δ*H*_*a*_^‡^ values.

On the other hand, during the dissociation of the thermodynamically stable BLF−κCG complex, the major contribution to Δ*H*_*d*_^‡^ is likely associated with conformational rearrangements of the binding sites. In the presence of KCl, electrostatic screening promotes the release of counterions associated with the electrical double layer, thereby altering the electrostatic interactions between charged segments of the biopolymers. Consequently, a greater energetic input is required to reorganize the binding sites during complex dissociation, resulting in positive Δ*H*_*d*_^‡^ values.

The negative *T*Δ*S*_*x*_^‡^ values for both the association and dissociation steps support this hypothesis. As discussed above, electrostatic screening by added salt promotes the release of counterions from the electrical double layer surrounding the biomolecules. Consequently, fewer counterions remain available for release during the formation of the [BLF – κCG]^‡^ complex, thereby reducing the favorable entropic contribution associated with counterion release and leading to more negative *T*Δ*S*_*x*_^‡^ values.

#### 3.2.2 Thermodynamic Study

To compare the surface-based thermodynamic parameters with those determined in solution by FS, *K*_*b*_^*SPR*^, Δ*G*_*SPR*_^*o*^, Δ*H*_SPR_^*o*^, and *T*Δ*S*_*SPR*_^*o*^ for BLF−κCG complex formation were determined by SPR. Based on the obtained kinetic constants (*k*_*a*_ and *k*_*d*_), the equilibrium binding constant was calculated using eq 13.^65^

13 $$K_{b}^{S\mathrm{PR}}=\frac{k_{a}}{k_{d}}$$

Additionally, the temperature dependence of *K*_*b*_^SPR^ (Figure S8) was used to calculate Δ*H* ^*o*^ using the van’t Hoff equation (eq 4). The values of Δ*G* ^*o*^ and *T*Δ*S* ^*o*^ were then calculated using eqs 3 and 5, respectively. The thermodynamic parameters for BLF−κCG complex formation determined by SPR at pH 4.0 and different temperatures are summarized in Table 5.

**Table 5:** Thermodynamic Parameters (Δ*G*_*SPR*_^*o*^, Δ*H*_SPR_^*o*^, and *T*Δ*S*_SPR_^*o*^) for BLF−κCG Complex Formation Determined by SPR at pH 4.0 in the Absence and Presence of KCl (100 mM)

***T***	***K*_*b*_^SPR^**	**Δ** *H*_SPR_^*o*^	**Δ** *G*_SPR_^*o*^	*T* **Δ** *S*_SPR_^*o*^
**K**	**10** ^**7**^ **M** ^**–1**^		**kJ mol^–1^**	
		**0 mM KCl**		
285.2	18.1 ± 0.1	–80.5 ± 0.9	–45.1 ± 0.9	–35.4 ± 0.9
289.2	9.2 ± 0.1	–80.5 ± 0.9	–44.1 ± 0.7	–36.4 ± 0.9
293.2	5.8 ± 0.1	–80.5 ± 0.9	–43.6 ± 0.7	–36.9 ± 0.9
297.2	3.9 ± 0.1	–80.5 ± 0.9	–43.2 ± 0.4	–37.4 ± 0.9
298.2	3.5 ± 0.1	–80.5 ± 0.9	–43.1 ± 0.6	–37.4 ± 0.9
301.2	3.0 ± 0.1	–80.5 ± 0.9	–43.1 ± 0.7	–37.4 ± 0.9
	**100 mM KCl**			
285.2	12.9 ± 0.2	–66.45 ± 0.9	–44.3 ± 0.5	–22.2 ± 0.9
289.2	8.4 ± 0.6	–66.45 ± 0.9	–43.9 ± 0.7	–22.6 ± 0.9
293.2	5.7 ± 0.3	–66.45 ± 0.9	–43.5 ± 0.6	–22.9 ± 0.9
297.2	4.3 ± 0.5	–66.45 ± 0.9	–43.4 ± 0.6	–23.0 ± 0.9
298.2	3.7 ± 0.3	–66.45 ± 0.9	–43.2 ± 0.7	–23.2 ± 0.9
301.2	2.8 ± 0.2	–66.45 ± 0.9	–43.9 ± 0.6	–23.5 ± 0.9

Although the binding constants determined by SPR were generally lower than those obtained by FS, both techniques revealed the same temperature dependence, with *K*_*b*_ decreasing as the temperature increased, indicating that BLF−κCG complex formation became less favorable at higher temperatures. Moreover, despite the differences in the *K*_*b*_ values, the corresponding Δ*G* ^◦^ values obtained by SPR were in good agreement with those determined by FS (−47.0 ≤ Δ*G*_FS_^*o*^ ≤ −46.2 kJ mol^–1^), confirming that the formation of the BLF−κCG complex was thermodynamically favorable relative to the free ligands (BLF and κCG) at equilibrium (i.e., BLF + κCG ⇌ BLF – κCG).

Although both techniques yielded negative values of Δ*H* ^*o*^ and *T*Δ*S* ^*o*^, their magnitudes differed. The enthalpy change determined by SPR (Δ*H*_SPR_^*o*^ = −80.5 kJ mol^–1^) was approximately 1.4 times more negative than that obtained by FS (Δ*H*_FS_^*o*^ = −57.4 kJ mol^–1^). Likewise, the entropic contribution determined by SPR (−37.4 ≤ *T*Δ*S*_SPR_^*o*^ ≤ −35.4 kJ mol^–1^) was consistently more negative than that obtained by FS (−11.3 ≤ *T*Δ*S*_FS_^*o*^ ≤ −10.4 kJ mol^–1^) over the entire temperature range investigated.

The differences in the thermodynamic parameters obtained by SPR and FS can be further rationalized by considering the distinct physical configurations and molecular observables associated with each technique. SPR provides an integrated response to κCG interactions occurring over the accessible surface of immobilized BLF, whereas intrinsic fluorescence spectroscopy is selectively sensitive to changes in the local environment of Trp residues arising from direct perturbation or binding-induced conformational changes.^66^ Thus, the more negative ΔH° obtained by SPR compared with FS may indicate that κCG establishes additional favorable interactions at BLF regions that are not efficiently reported by the Trp fluorescence response. In addition, BLF immobilization in SPR restricts the translational and rotational freedom of the protein and changes the dimensionality and configurational constraints of the binding reaction. Such restrictions can alter the entropy associated with complex formation relative to interactions between freely diffusing molecules in solution. Indeed, comparative studies have demonstrated that immobilization in SPR can produce different entropy contributions from those obtained using solution-phase fluorescence measurements, even when the same molecular interaction is investigated.^67^ Therefore, the more negative ΔS° obtained by SPR may reflect, at least in part, the different configurational constraints imposed by protein immobilization.

##### 3.2.2.1 Effect of Ionic Strength on the Formation of the Thermodynamically Stable BLF−κCG Complex

Protein–polysaccharide interactions are governed by a variety of intermolecular forces, including electrostatic interactions, hydrogen bonding, van der Waals forces, and hydrophobic interactions.^57^ Consequently, these interactions can be modulated by changes in pH, ionic strength, and the dielectric constant of the solvent.

To investigate the effect of ionic strength on the thermodynamics of BLF−κCG complex formation, the *K*_*b*_^SPR^, Δ*G*_SPR_^*o*^, Δ*H*_SPR_^*o*^, and *T*Δ*S*_SPR_^*o*^ were determined in the presence of KCl (100 mM) using eqs 13, 3, 4, and 5, respectively. The van’t Hoff plots for the interaction between BLF and κCG in the presence of KCl are shown in Figure S9. The corresponding values of *K*_*b*_^SPR^, Δ*G*_SPR_^*o*^, Δ*H*_SPR_^*o*^, and *T*Δ*S*_SPR_^*o*^ at different temperatures are summarized in Table 5.

Increasing the ionic strength of the medium resulted in lower *K*_*b*_^SPR^ values and, consequently, less negative Δ*G*_SPR_^*o*^ values. In addition, the presence of KCl caused both Δ*H*_SPR_^*o*^ and *T*Δ*S*_SPR_^*o*^ to become less negative. As previously discussed for the kinetic parameters, the addition of salt promotes the shielding of electrostatic interactions and the subsequent release of counterions from the electrical double layers surrounding BLF and κCG. Under these conditions, the formation of the BLF−κCG complex occurs through the approach of positively charged regions of BLF and negatively charged sulfated groups of κCG, leading to favorable electrostatic attraction between the interacting species. As a result, the electrostatic contribution to complex formation becomes less favorable with increasing ionic strength, yielding less negative Δ*H*_SPR_^*o*^ values. Simultaneously, the shielding of intramolecular electrostatic interactions within the biopolymers increases the conformational flexibility of the BLF and κCG binding sites, resulting in less negative *T*Δ*S*_SPR_^*o*^ values.

## 4 Conclusions

Both SPR and FS analyses demonstrated that BLF interacts with κCG to form a 1:1 complex in aqueous solution at pH 4.0. This interaction is primarily driven by favorable intermolecular interactions, including electrostatic attraction, van der Waals forces, and hydrogen bonding. Although increasing the ionic strength promotes the shielding of electrostatic interactions, the thermodynamic stability of the BLF−κCG complex remains essentially unchanged. However, the increase in ionic strength decreases the values of Δ*H*_*x*_^‡^ and *T*Δ*S*_*x*_^‡^ for both the association and dissociation processes, indicating that ionic shielding influences not only the interaction energy but also the molecular configuration of the transition states involved in complex formation and dissociation.

Furthermore, SPR kinetic analyses revealed that BLF−κCG complex formation proceeds through a multistep mechanism involving a transition-state complex ([BLF – κCG]^‡^). The association of free BLF and κCG molecules to form [BLF – κCG]^‡^ occurs more rapidly than the dissociation of the thermodynamically stable BLF−κCG complex, as evidenced by Δ*G*_*a*_^‡^ < Δ*G*_*d*_^‡^ despite Δ*H*_*a*_^‡^ > Δ*H*_*d*_^‡^ and *T*Δ*S*_*a*_^‡^ > *T*Δ*S*_*d*_^‡^. These results indicate that molecular desolvation makes a major contribution to the faster association process. Moreover, increasing the ionic strength reduced the differences between the enthalpic and entropic activation parameters for association and dissociation, leading to a transition toward an effectively single-step mechanism. This behavior is likely a consequence of electrostatic shielding of the macromolecular electrical double layers and the consequent release of counterions from these regions.

The kinetic and thermodynamic parameters obtained provide a molecular basis for evaluating the stability, persistence, and temperature dependence of the BLF-κCG complex. By defining the rates of complex formation and dissociation, equilibrium affinity, and energetic contributions governing association, these parameters can guide the selection of formulation and processing conditions that favor complex stability or controlled dissociation. Such information can therefore support the rational development of protein–polysaccharide systems for potential applications in bioactive compound encapsulation and delivery, emulsion stabilization, and functional food formulations.

## Supplementary Material



## Notes

Figures showing the Stern–Volmer plots used for determination of the interaction parameters between native BLF and κCG and double logarithmic models at different temperatures (Figure S1); van’t Hoff plot for the determination of the dependence of *K*_*b*_^FS^ on temperature (Figure S2); sensorgrams for BLF interacting with different concentrations of κCG at various temperatures (Figure S3); plot of *k*_obs_ as a function of κCG concentration used to determine *k*_*a*_ at different temperatures (Figure S4); sensorgrams for BLF interacting with different concentrations of κCG in the presence of KCl at various temperatures (Figure S5); plot of *k*_obs_ as a function of κCG concentration in the presence of KCl used to determine *k*_*a*_ at different temperatures (Figure S6); Arrhenius plots associated with the BLF−κCG interaction in the presence of the KCl (Figure S7); van’t Hoff plot (ln *K*_*b*_^SPR^ as a function of 1/*T*) for the interaction between BLF and κCG obtained by SPR (Figure S8); van’t Hoff plot (ln *K*_*b*_^SPR^ as a function of 1/*T*) for the interaction between BLF and κCG in the presence of KCl studied by SPR (Figure S9); Table S1 contains the Stern–Volmer constants (*K*_SV_) and bimolecular quenching rate constant (*k*_q_) of BLF−κCG complex formation at different temperatures; Table S2 contains the hydrodynamic diameter (*D*_*H*_) and Zeta potential (ζ) values for free BLF, free κCG, and BLF−κCG mixtures containing different κCG concentrations; and Table S3 contains the activation parameters for the formation of the transition complex (PDF)

## §

Colloid Chemistry Group, Institute of Chemistry, Federal University of Alfenas, Rua Gabriel Monteiro da Silva, 700, Alfenas, MG 37130–000, Brazil

## ∥

Department of Physical Chemistry, Institute of Chemistry, University of Campinas (UNICAMP), Campinas, SP 13083–970, Brazil.

## ⊥

Department of Chemistry, Federal University of Oeste do Pará, Santarém, PA 68040–070, Brazil.
