# Supplementary material for: Lactoferrin/κ-Carrageenan Interactions: Thermodynamic and Kinetic Insights through Surface Plasmon Resonance and Steady-State Fluorescence
Source: ACS Omega. 2026 Sep 26;11(39):60360–70. doi: 10.1021/acsomega.6c08383 (PMC13647915; doi:10.1021/acsomega.6c08383)
Supplement: Supplementary file 1 [file ao6c08383_si_001.pdf]

Supplementary Material

Lactoferrin/κ-carrageenan Interactions: Thermodynamic and Kinetic Insights through Surface Plasmon Resonance and Steady-State Fluorescence

Yara Luiza Coelho Zampier[1,†], Isabela A. Marques[1], Álvaro Javier Patiño-Agudelo[1,‡], Hauster Maximiler C. de Paula[1,§], Eliara A. Hudson[2], Ana Clarissa S. Pires[2], Luis Henrique M. da Silva[1]*

[1]Advanced Thermokinetics of Molecular Systems (ATOMS) Group, Chemistry Department, Federal University of Viçosa, PH Rolfs Avenue, 36570-000 Viçosa-MG, Brazil.

[2]Department of Food Technology, Federal University of Viçosa, Av. P. H. Rolfs s/s, 36570900, Viçosa-MG, Brazil.

† Present Address: Colloid Chemistry Group, Institute of Chemistry, Federal University of Alfenas, Rua Gabriel Monteiro da Silva, 700, Alfenas, MG 37130-000, Brazil.

‡ Present Address: Department of Physical Chemistry, Institute of Chemistry, University of Campinas (UNICAMP), Campinas, SP 13083-970, Brazil.

§ Present Address: Department of Chemistry, Federal University of Oeste do Pará, Santarém, PA 68040-070, Brazil.

E-mails:
yara.coelho@unifal-mg.edu.br
marques.isabela100@gmail.com
patinoagudeloaj@gmail.com
hauster.campos@gmail.com
eliara.hudson@ufv.br
anaclarissasp@yahoo.com.br

*Corresponding author: luhen@ufv.br; Phone: +55 31 38993052; Fax: +55 31 38992175

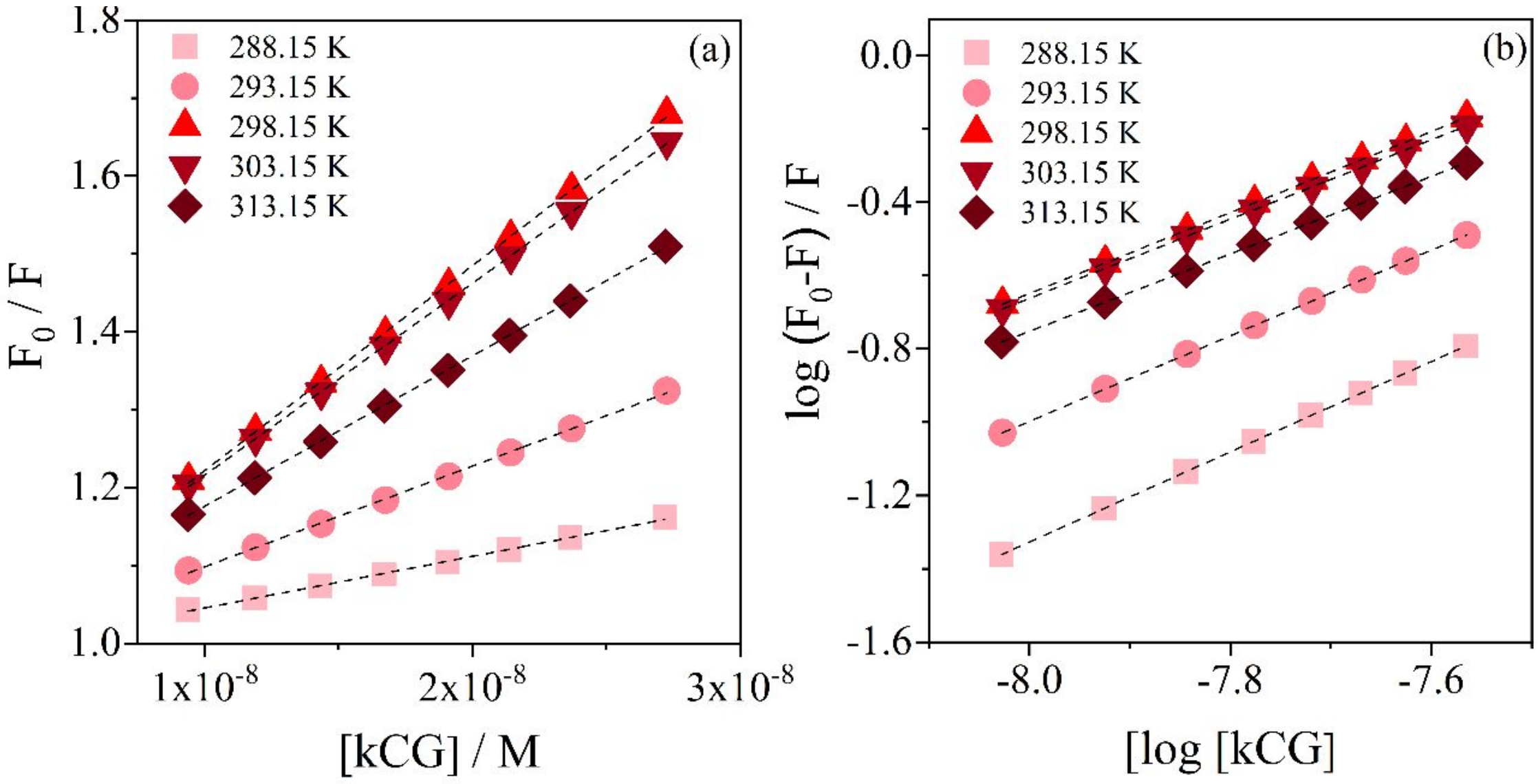

Figure S1. Plots for determination of the interaction parameters between native BLF and κCG according to the **(a)** Stern-Volmer and **(b)** double-logarithmic models, obtained at different temperatures and pH 4.0.

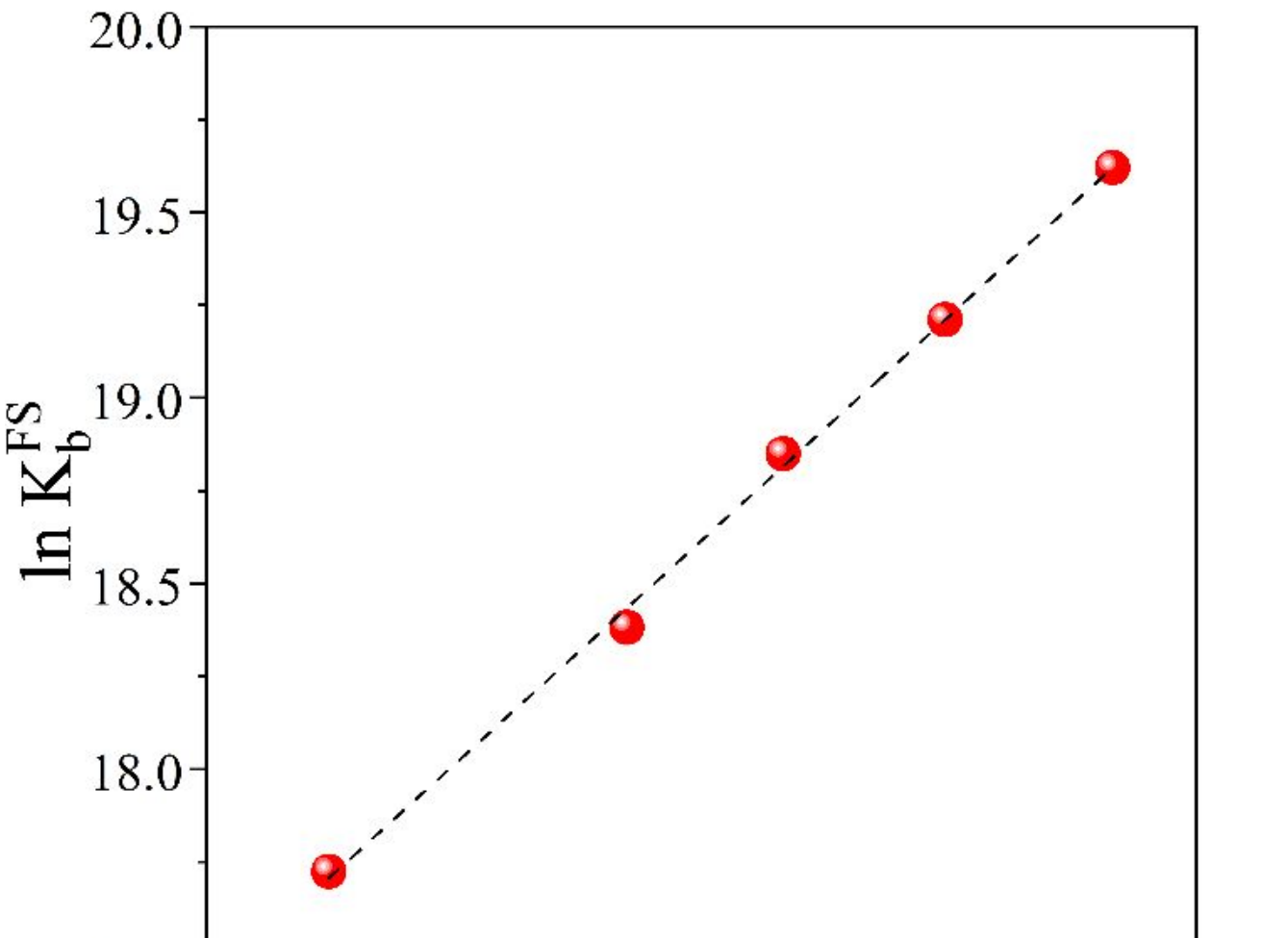

Figure S2. van't Hoff plot (ln K_b^{FS} versus 1/T) for the interaction between BLF and κCG.

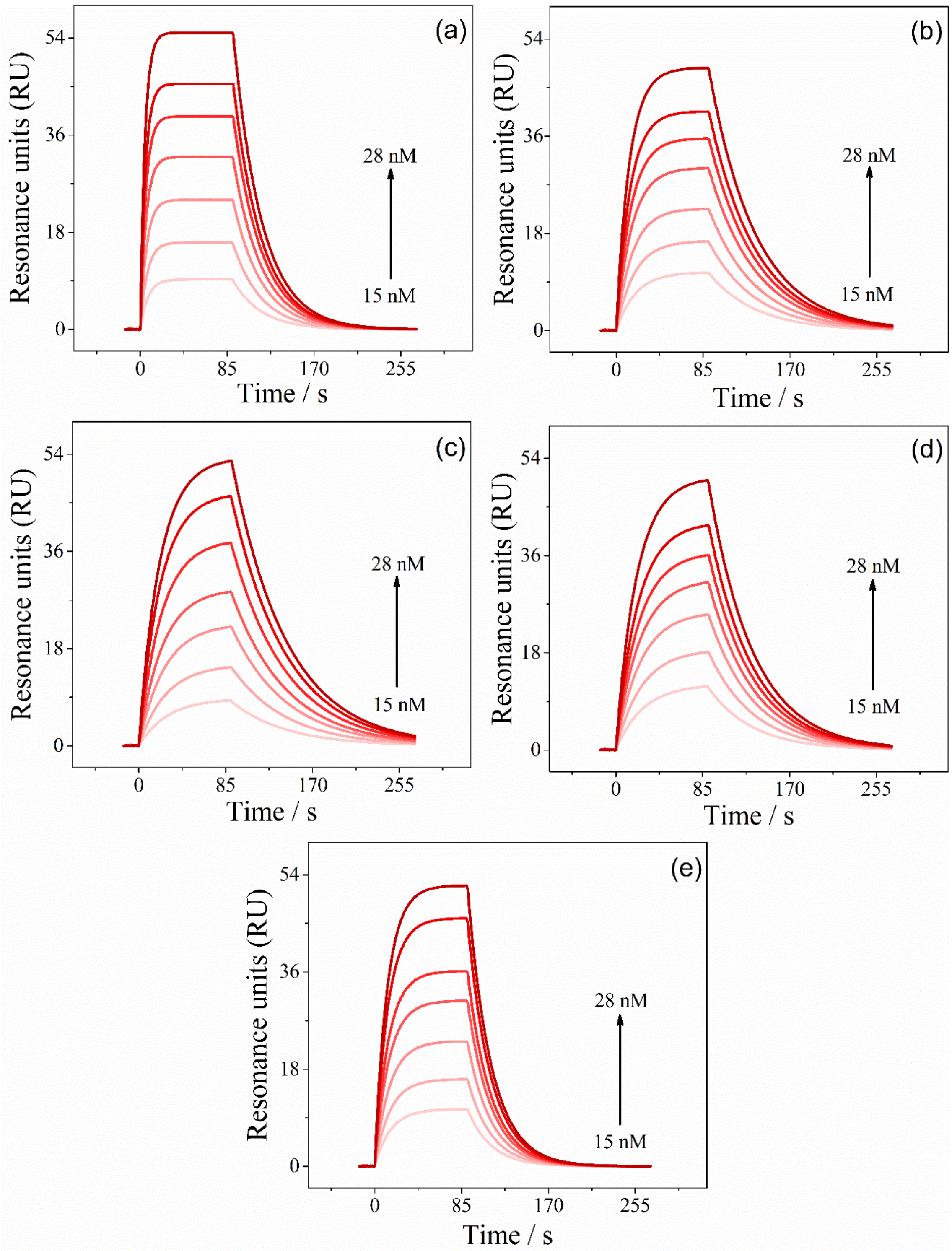

Figure S3. SPR sensorgrams for the interaction between BLF and κCG at different κCG concentrations (15-28 nM), pH 4.0, and different temperatures: **(a)** 285.2 K, **(b)** 289.2 K, **(c)** 293.2 K, **(d)** 297.2 K, and **(e)** 301.2 K.

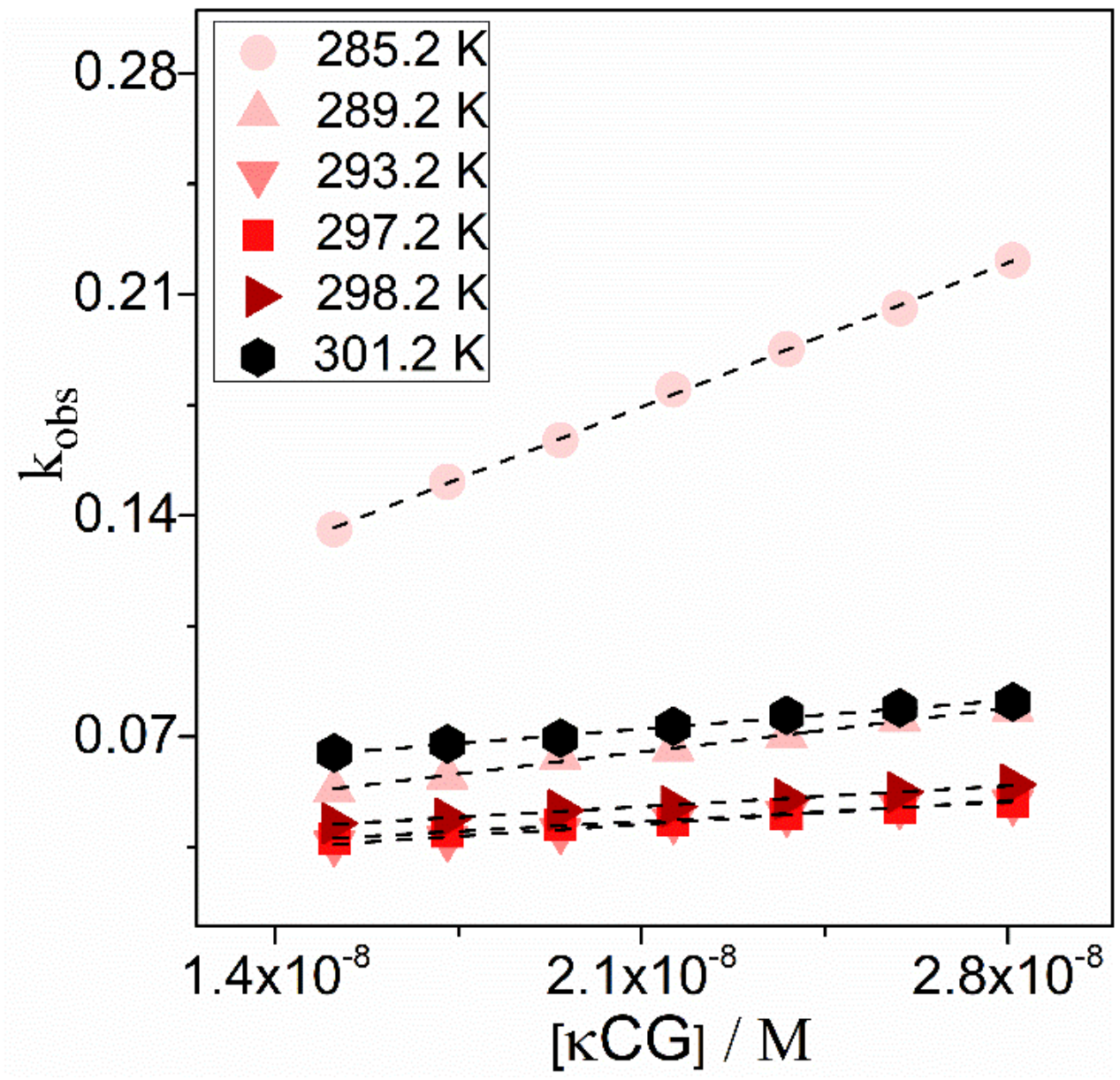

Figure S4. Plot of k_{obs} as a function of κCG concentration used to determine k_a at temperatures and pH 4.0.

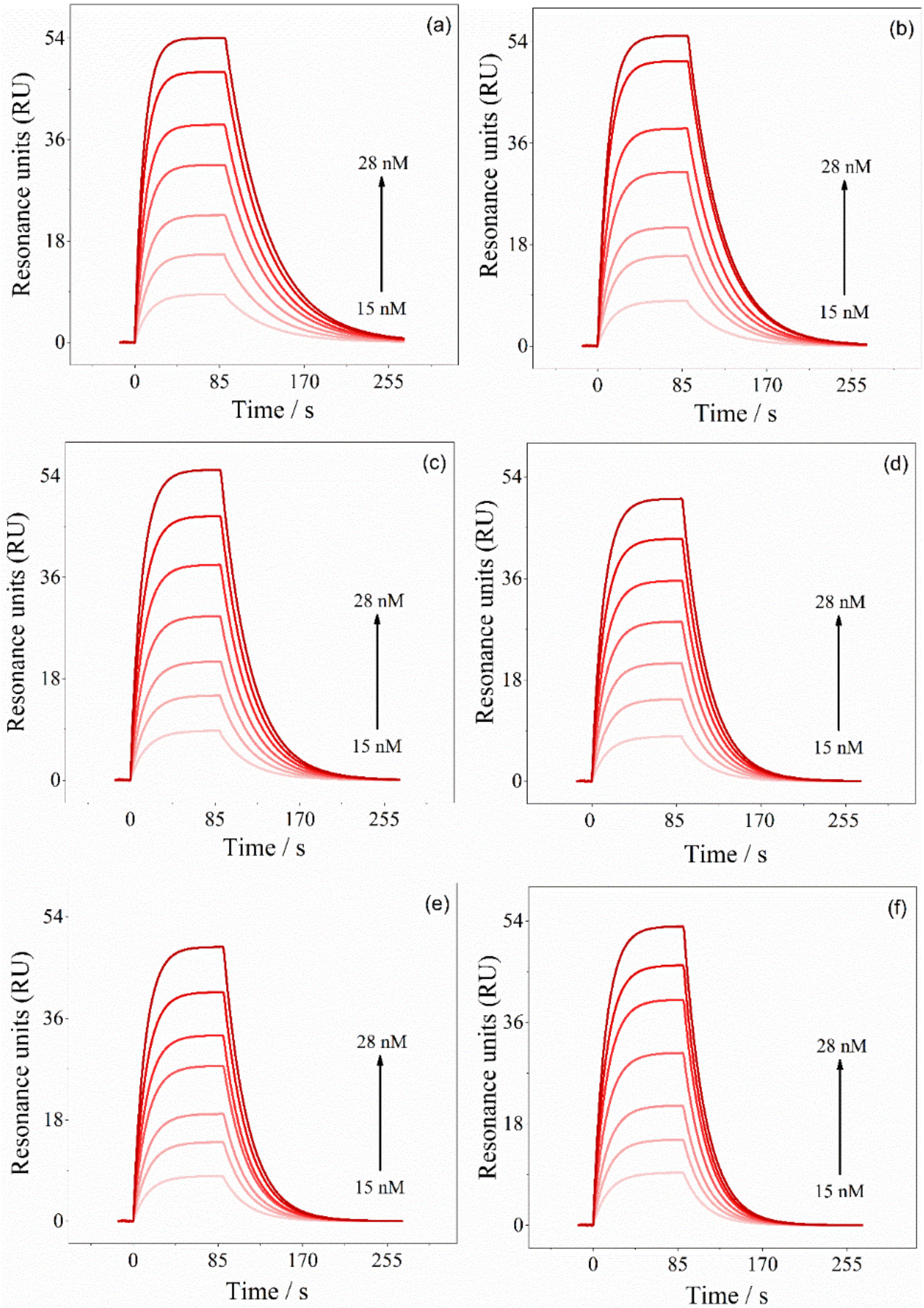

Figure S5. SPR sensorgrams for the interaction between BLF and κCG at different κCG concentrations (15-28 nM) in the presence of the 100 mM KCl (pH 4.0) at different temperatures: **(a)** 285.2 K, **(b)** 289.2 K, **(c)** 293.2 K, **(d)** 297.2 K, **(e)** 298.2 K, and **(f)** 301.2 K.

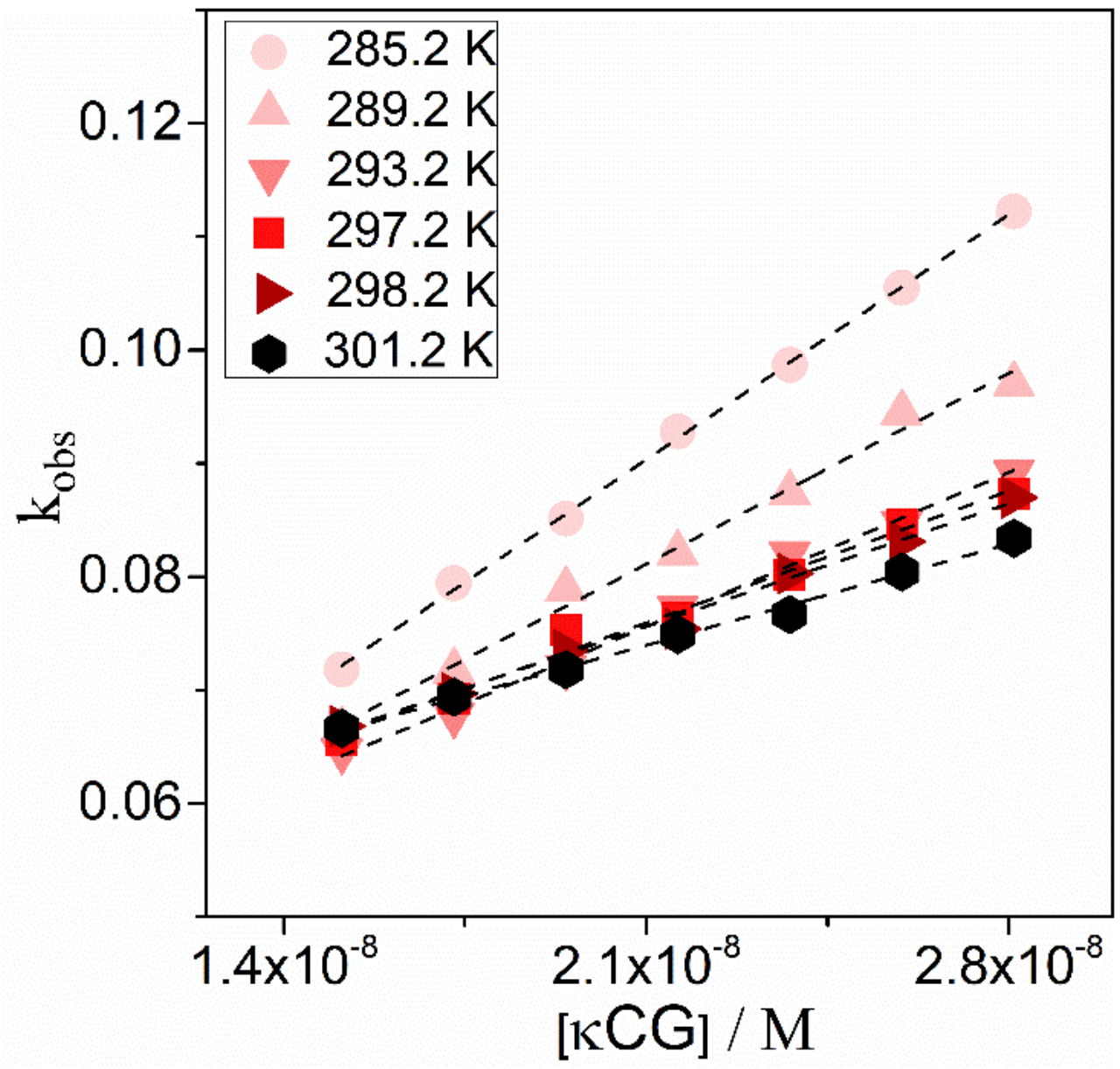

Figure S6. Plot of k_{obs} as a function of κCG concentration, used to determine the association rate constant (k_a), in the presence of 100 mM KCl at pH 4.0.

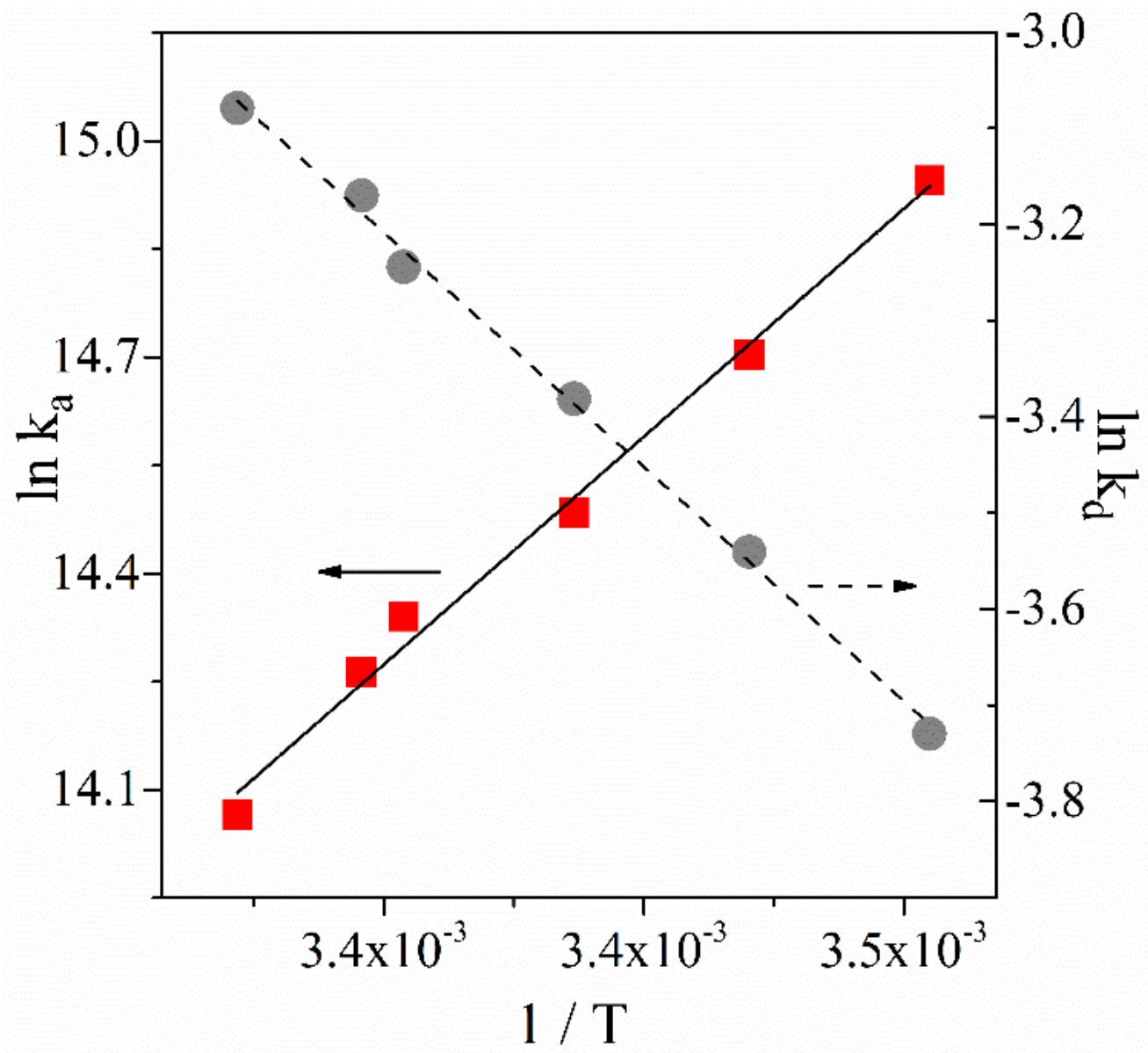

Figure S7. Arrhenius plots of $ln\ k_a$ (■) and $ln\ k_d$ (●) *versus* $1/T$ for the BLF-κCG interaction in the presence of 100mM KCl at pH 4.0.

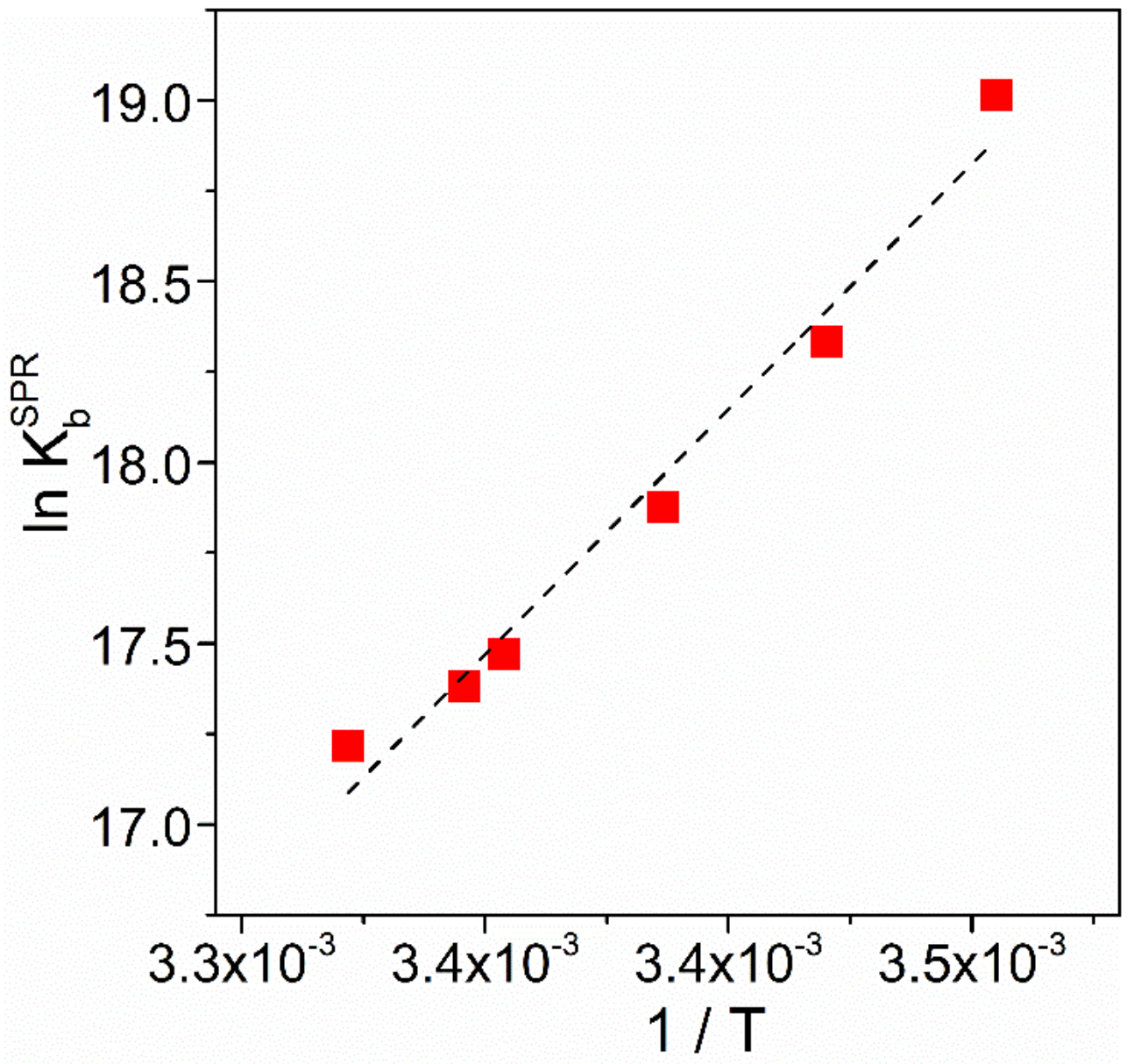

Figure S8. van't Hoff plot (ln K_b^{SPR} versus $1/T$) for the interaction between BLF and κCG determined by SPR at pH 4.0.

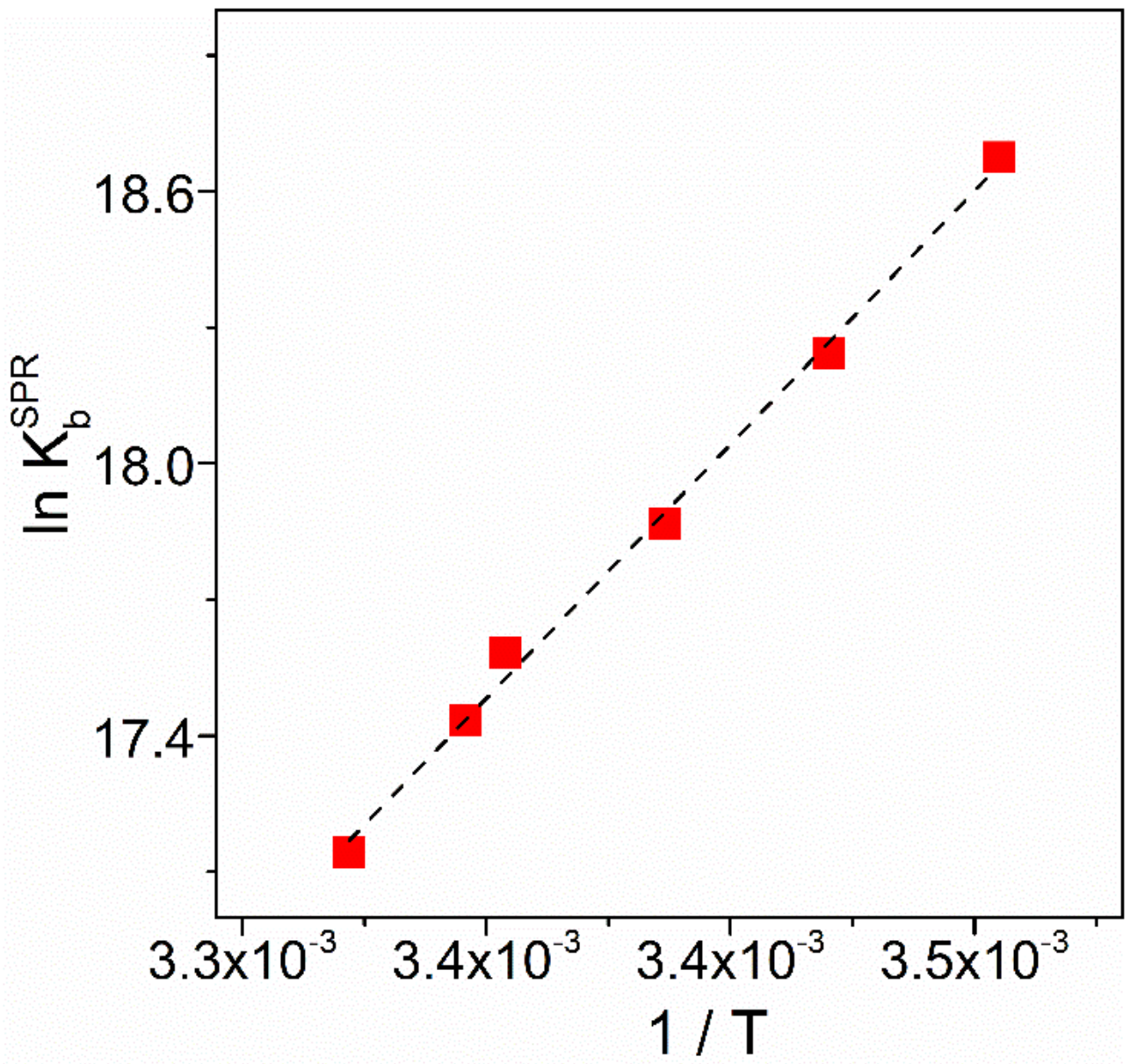

Figure S9. van't Hoff plot (ln K_b^{SPR} versus $1/T$) for the interaction between BLF and κCG determined by SPR in the presence of the KCl (100 mM) and pH 4.0.

Table S1. Stern-Volmer constant (K_{SV}) and bimolecular quenching rate constant (k_{q}) for the interaction between BLF and κCG at different temperatures (T) and pH 4.0.

$\boldsymbol{T}$	$\boldsymbol{K_{\mathrm{SV}}}$	$\boldsymbol{k_{\mathrm{q}}}$
K	10^7 M^{-1}	10^{16} $M^{-1}s^{-1}$
288.2	0.66 ± 0.01	0.62 ± 0.01
293.2	1.29 ± 0.01	1.22 ± 0.01
298.2	2.63 ± 0.02	2.48 ± 0.02
303.2	2.47 ± 0.01	2.33 ± 0.01
313.2	1.93 ± 0.07	1.82 ± 0.01

Table S2. Hydrodynamic diameter (D_H) and Zeta potential (ζ) of free BLF, free κCG and BLF-κCG mixtures containing different κCG concentrations at pH 4.0 and 298.2 K.

Sample	$\boldsymbol{D_H}$ / nm	$\boldsymbol{\zeta}$ / mV
**BLF 20 µM**	72 ± 1	14 ± 2
**κCG 1.1 nM**	601 ± 51	5 ± 1
**κCG 35 nM**	503 ± 54	5 ± 1
**κCG 85 nM**	585 ± 29	5 ± 2
**BLF 20 µM + κCG 1.1 nM**	143 ± 8	20 ± 1
**BLF 20 µM + κCG 35 nM**	260 ± 2	23 ± 1
**BLF 20 µM + κCG 85 nM**	278 ± 6	23 ± 1

Table S3. Activation parameters for the formation of the transition complex through (a) association of free BLF and κCG molecules and (d) dissociation of the thermodynamically stable BLF–κCG complex at different temperatures and pH 4.0, at KCl concentrations of 1 and 10 mM.

T	Association step (a)				Dissociation step (d)			
	$E_a^{\ddagger}$	$\Delta H_a^{\ddagger}$	$\Delta G_a^{\ddagger}$	$T\Delta S_a^{\ddagger}$	$E_d^{\ddagger}$	$\Delta H_d^{\ddagger}$	$\Delta G_d^{\ddagger}$	$T\Delta S_d^{\ddagger}$
**K**	**kJ mol^{-1}**							
				**1 mM KCl**				
285.2	-53.4 ± 0.9	55.7 ± 0.9	48.6 ± 0.1	-104.4 ± 0.8	20.7 ± 0.1	18.3 ± 0.1	79.3 ± 0.1	-60.9 ± 0.1
289.2	-53.4 ± 0.9	55.8 ± 0.9	49.9 ± 0.1	-105.7 ± 0.8	20.7 ± 0.1	18.3 ± 0.1	80.1 ± 0.1	-61.8 ± 0.1
293.2	-53.4 ± 0.9	55.8 ± 0.9	51.4 ± 0.1	-107.2 ± 0.8	20.7 ± 0.1	18.3 ± 0.1	80.9 ± 0.1	-62.7 ± 0.1
297.2	-53.4 ± 0.9	55.8 ± 0.9	53.0 ± 0.1	-108.8 ± 0.8	20.7 ± 0.1	18.2 ± 0.1	81.8 ± 0.1	-63.6 ± 0.1
298.2	-53.4 ± 0.9	55.8 ± 0.9	53.3 ± 0.1	-109.2 ± 0.8	20.7 ± 0.1	18.2 ± 0.1	82.0 ± 0.1	-63.8 ± 0.2
301.2	-53.4 ± 0.9	55.9 ± 0.9	54.4 ± 0.1	-110.3 ± 0.8	20.7 ± 0.1	18.2 ± 0.2	82.6 ± 0.1	-64.4 ± 0.2
				**10 mM KCl**				
285.2	-58.2 ± 0.9	-60.6 ± 0.5	48.3 ± 0.9	-108.9 ± 0.9	20.8 ± 0.8	18.4 ± 0.5	79.8 ± 0.9	-61.4 ± 0.9
289.2	-58.2 ± 0.9	-60.6 ± 0.5	49.6 ± 0.9	-110.2 ± 0.9	20.8 ± 0.8	18.4 ± 0.5	80.7 ± 0.9	-62.3 ± 0.9
293.2	-58.2 ± 0.9	-60.6 ± 0.5	51.0 ± 0.9	-111.6 ± 0.9	20.8 ± 0.8	18.4 ± 0.5	81.5 ± 0.9	-63.2 ± 0.9
297.2	-58.2 ± 0.9	-60.7 ± 0.5	52.8 ± 0.9	-113.5 ± 0.9	20.8 ± 0.8	18.3 ± 0.5	82.4 ± 0.9	-64.1 ± 0.9
298.2	-58.2 ± 0.9	-60.7 ± 0.5	53.2 ± 0.9	-113.9 ± 0.9	20.8 ± 0.8	18.3 ± 0.5	82.6 ± 0.9	-64.3 ± 0.9
301.2	-58.2 ± 0.9	-60.7 ± 0.5	54.3 ± 0.9	-115.0 ± 0.9	20.8 ± 0.8	18.3 ± 0.5	83.2 ± 0.9	-65.0 ± 0.9
